# Supplementary material for: Grasp55−/− mice display impaired fat absorption and resistance to high-fat diet-induced obesity
Source: Nat Commun. 2020 Mar 17;11:1418. doi: 10.1038/s41467-020-14912-x (PMC7078302; doi:10.1038/s41467-020-14912-x)
Supplement: Supplementary file 1 — Supplementary Information [file 41467_2020_14912_MOESM1_ESM.pdf]

SUPPLEMENTARY INFORMATION

Grasp55$^{-/-}$ mice display impaired fat absorption and resistance to high-fat diet-induced obesity

Jiyoon Kim[1,2], Hyeyon Kim[3], Shin Hye Noh[1], Dong Geon Jang[1], Shi-Young Park[4], Dongkook Min[5], Hyunki Kim[6], Hee-Seok Kweon[7], Hoguen Kim[8], Sowon Aum[1], Sookyung Seo[1], Cheol Soo Choi[4], Hail Kim[6], Jae Woo Kim[5], Seok Jun Moon[3], Heon Yung Gee[1], Min Goo Lee[1,*]

[1]Department of Pharmacology, Brain Korea 21 Project for Medical Sciences, Severance Biomedical Science Institute, Yonsei University College of Medicine, Seoul 03722, Korea. [2]Department of Pharmacology, Department of Biomedicine & Health Sciences, College of Medicine, The Catholic University of Korea, Seoul, 06591, Korea. [3]Department of Oral Biology, BK21 PLUS, Yonsei University College of Dentistry, Yonsei-ro 50-1, Seodaemun-gu, Seoul 03722, Korea. [4]Korea Mouse Metabolic Phenotyping Center, Lee Gil Ya Cancer and Diabetes Institute, and Department of Internal Medicine, Gachon University College of Medicine, Incheon 21999, Korea. [5]Department of Biochemistry and Molecular Biology, Severance Biomedical Science Institute, Yonsei University College of Medicine, Seoul 03722, Korea. [6]Graduate School of Medical Science and Engineering, Korea Advanced Institute of Science and Technology, Daejeon 34141, Korea. [7]Center for Research Equipment, Korea Basic Science Institute, Cheongju 28119, Korea. [8]Department of Pathology, Brain Korea 21 Project for Medical Sciences, Yonsei University College of Medicine, Seoul 03722, Korea.

Supplementary Figures 1-22.

Supplementary Tables 1-5.

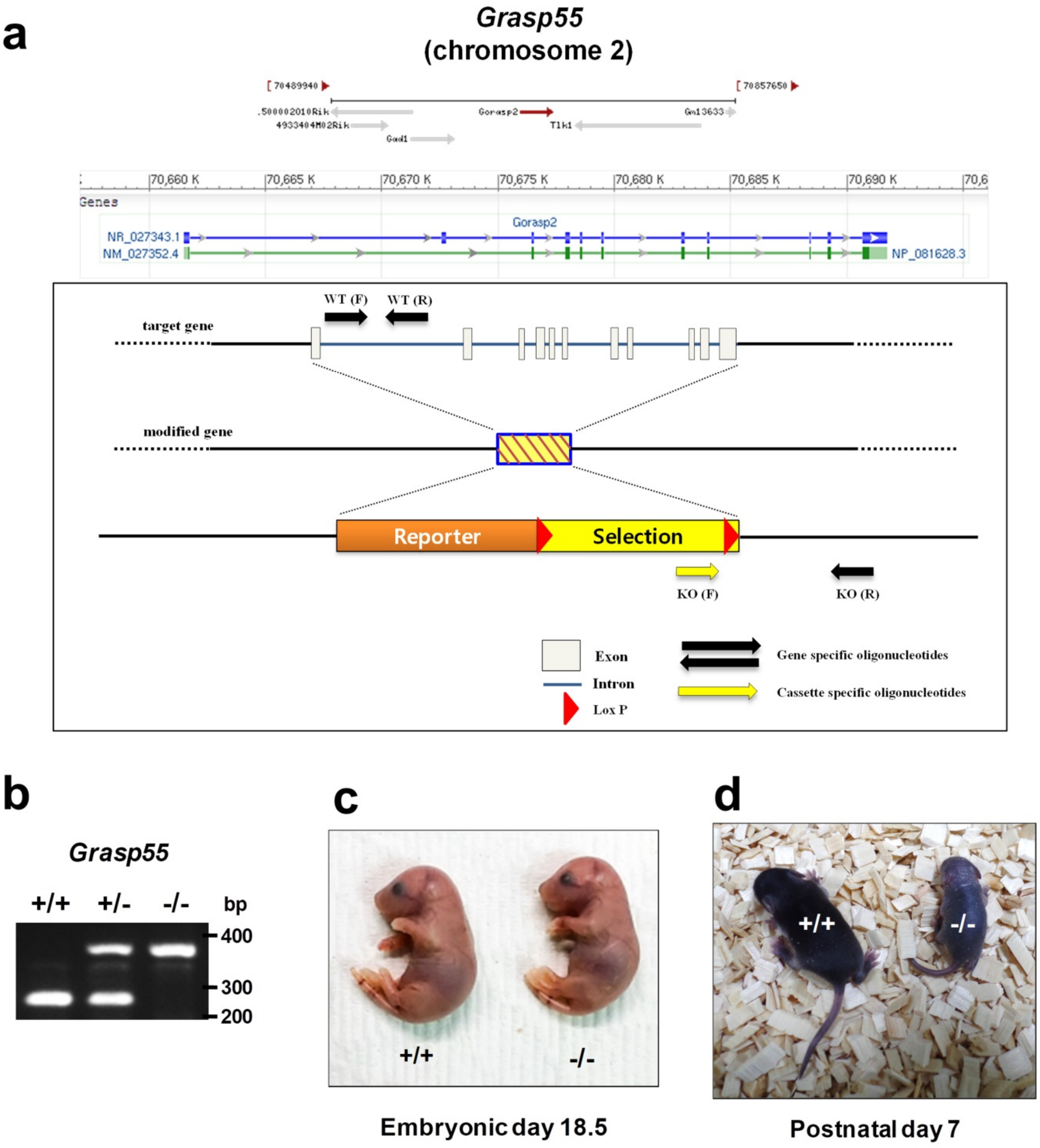

Supplementary Fig. 1 | *Grasp55*$^{-/-}$ mice have growth retardation. a, Schematic diagram of the strategy used to generate *Grasp55*$^{-/-}$ mice (Gorasp2$^{tm1(KOMP)Vlcg}$, strain; KOMP Repository, Oakland, CA, USA). The genotyping primer regions are marked with black and yellow arrows. **b**, PCR genotyping of genomic DNA samples from mice generated by heterozygote (+/-) crosses. **c**, **d**, Photographs of wild-type (*Grasp55*$^{+/+}$) and *Grasp55*$^{-/-}$ mice at embryonic day 18.5 (**c**), postnatal day 7 (**d**). Source data are provided as a Source Data file.

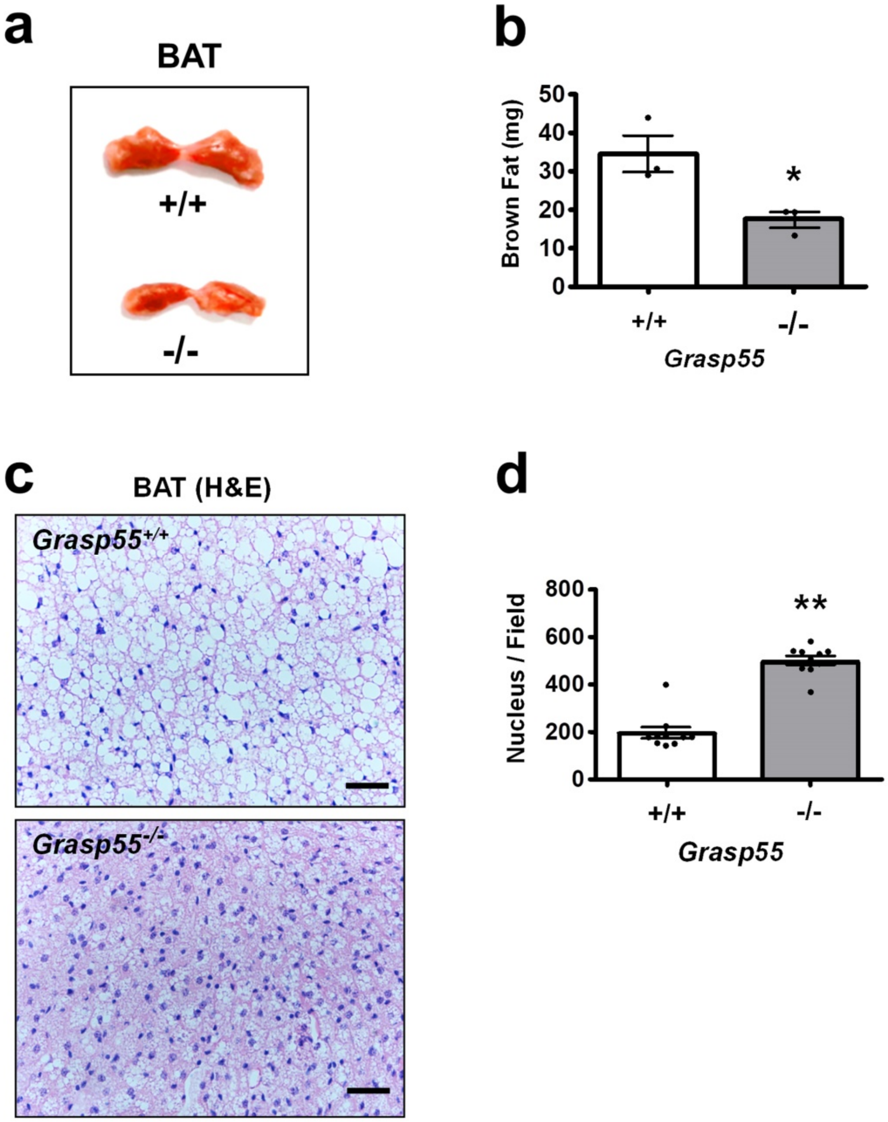

Supplementary Fig. 2 | Brown adipose tissues are reduced in *Grasp55*$^{-/-}$ mice. a, **b**, Photograph (**a**) and weight (**b**) of brown adipose tissue (BAT) of *Grasp55*$^{+/+}$ and *Grasp55*$^{-/-}$ mice at 4 weeks of age (n = 6). **c**, **d**, Histologic examination of BAT (H&E staining). Representative light microscopic tissue images are presented in (**c**). Quantitative analyses of the H&E images, that inversely correlate with fat contents of BAT by counting the number of adipocyte nuclei in a light microscopic field (450 × 340 μm), are depicted in (**d,** n = 10). Data are shown as mean ± SEM. Scale bars: 50 μm. $*p < 0.05$, $**p < 0.01$. All p values were calculated by unpaired two-tailed Student's t tests. Source data are provided as a Source Data file.

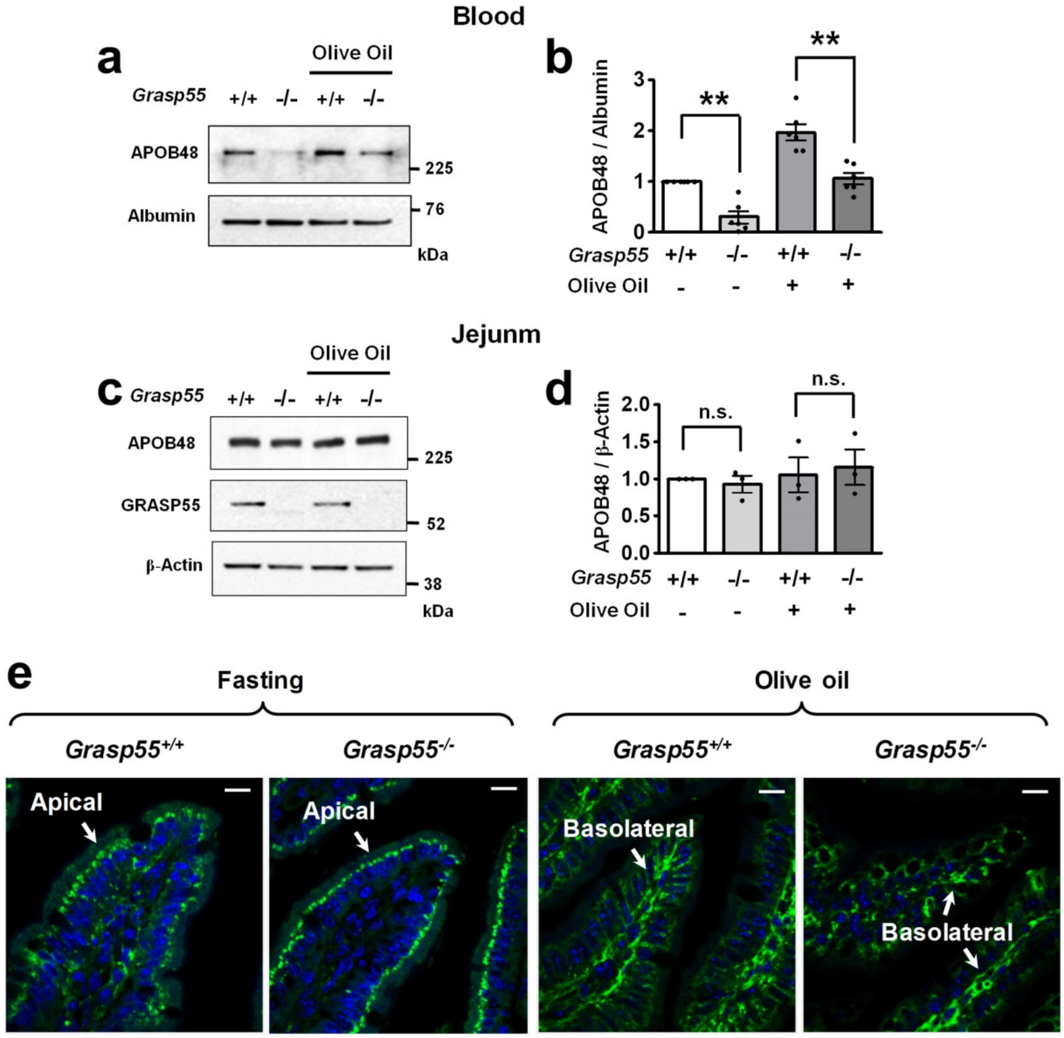

Supplementary Fig. 3 | GRASP55 deficiency reduces apolipoprotein B (APOB) levels in the blood, but not in the jejunum. a–d, Plasma apolipoprotein B48 (APOB48, **a**, **b**) and jejunal APOB48 (**c**, **d**) were identified by immunoblot. Samples were taken from *Grasp55*$^{+/+}$ and *Grasp55*$^{-/-}$ mice that were fasted for 16 h or 2 h after an oil bolus (olive oil 10 μl/g of body weight). Representative immunoblots are shown in (**a**, **c**) and summaries of multiple experiments are depicted in (**b**, **d**, n = 6 for each). Amounts of albumin and β-actin were monitored as protein loading controls for blood plasma and jejunal cells, respectively. Data are shown as mean ± SEM. n.s.: not significant, $^{**}p < 0.01$. All *p* values were calculated by unpaired two-tailed Student's *t* tests. Unprocessed blots can be found in Supplementary Fig. 22. **e**, Localization of APOB was examined by immunostaining with anti-APOB antibodies (green) in jejunal epithelia from *Grasp55*$^{+/+}$ and *Grasp55*$^{-/-}$ mice that were fasted for 16 or 4 h after oral gavage with olive oil (10 μl/g of body weight). Nuclei were counterstained with 4′,6-diamidino-2-phenylindole (blue). APOB proteins were concentrated in the subapical area of both *Grasp55*$^{+/+}$ and *Grasp55*$^{-/-}$ enterocytes under fasting conditions (left). Most APOB proteins were translocalized to the basolateral area after olive oil feeding in both *Grasp55*$^{+/+}$ and *Grasp55*$^{-/-}$ enterocytes (right). Note that APOBs in *Grasp55*$^{+/+}$ enterocytes are more highly concentrated near the basolateral membrane after olive oil feeding. Scale bars: 20 μm. Source data are provided as a Source Data file.

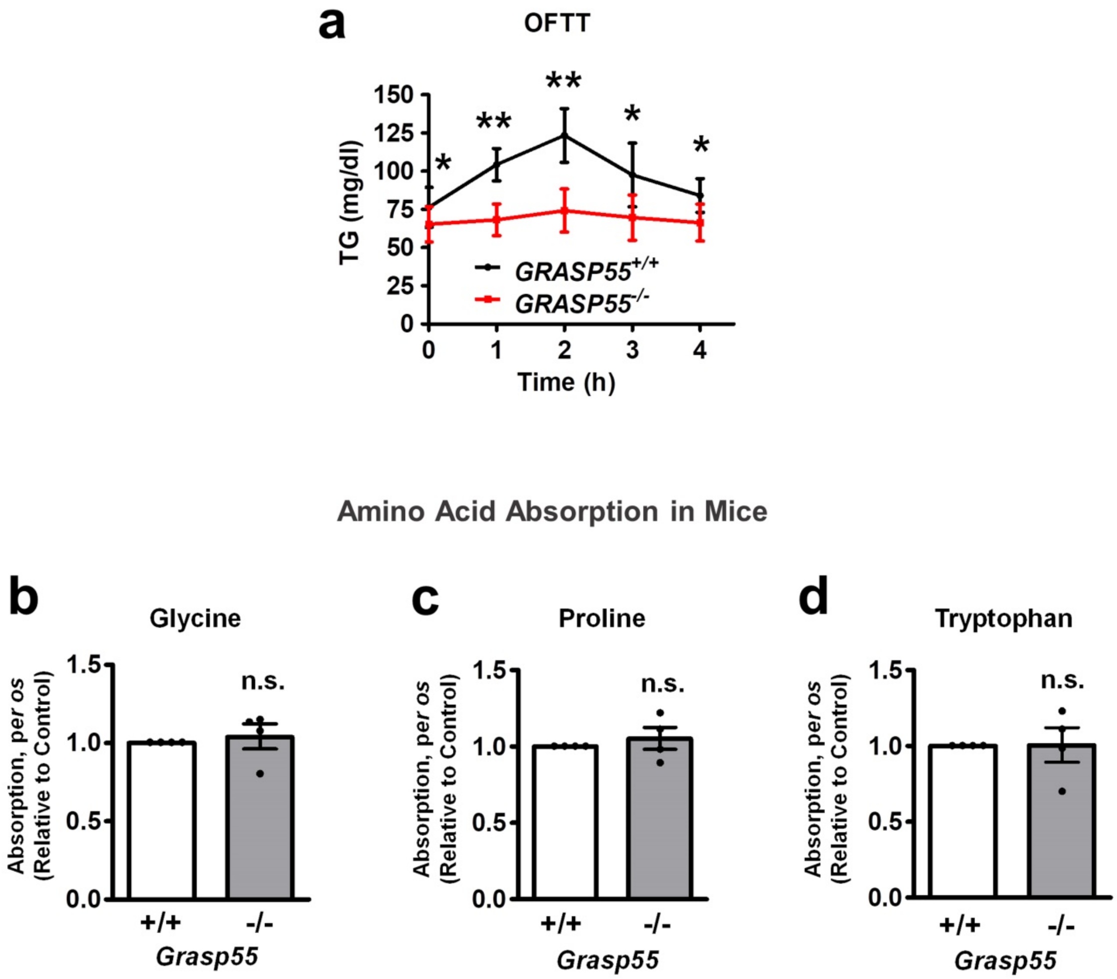

Supplementary Fig. 4 | GRASP55 deficiency reduces dietary lipid absorption but does not affect amino acid absorption in mice. a, An oral fat tolerance test (OFTT) was performed in *Grasp55*$^{+/+}$ and *Grasp55*$^{-/-}$ mice without tyloxapol treatment. Mice received oral gavage with olive oil (10 µl/g of body weight) after 16 h fasting. Triglyceride (TG) concentrations were determined in blood plasma (n = 6). **b–d**, Intestinal absorption of amino acids was measured in *Grasp55*$^{+/+}$ and *Grasp55*$^{-/-}$ mice at the age of 12 weeks using ^{14}C-radiolabeled glycine (**b**), L-proline (**c**), and L-tryptophan (**d**) as described in Methods (each n = 4). Data are shown as mean ± SEM. n.s.: not significant, *$p < 0.05$, **$p < 0.01$. P values were calculated by unpaired (**a**) or paired (**b**, **c** and **d**) two-tailed Student's t tests. Source data are provided as a Source Data file.

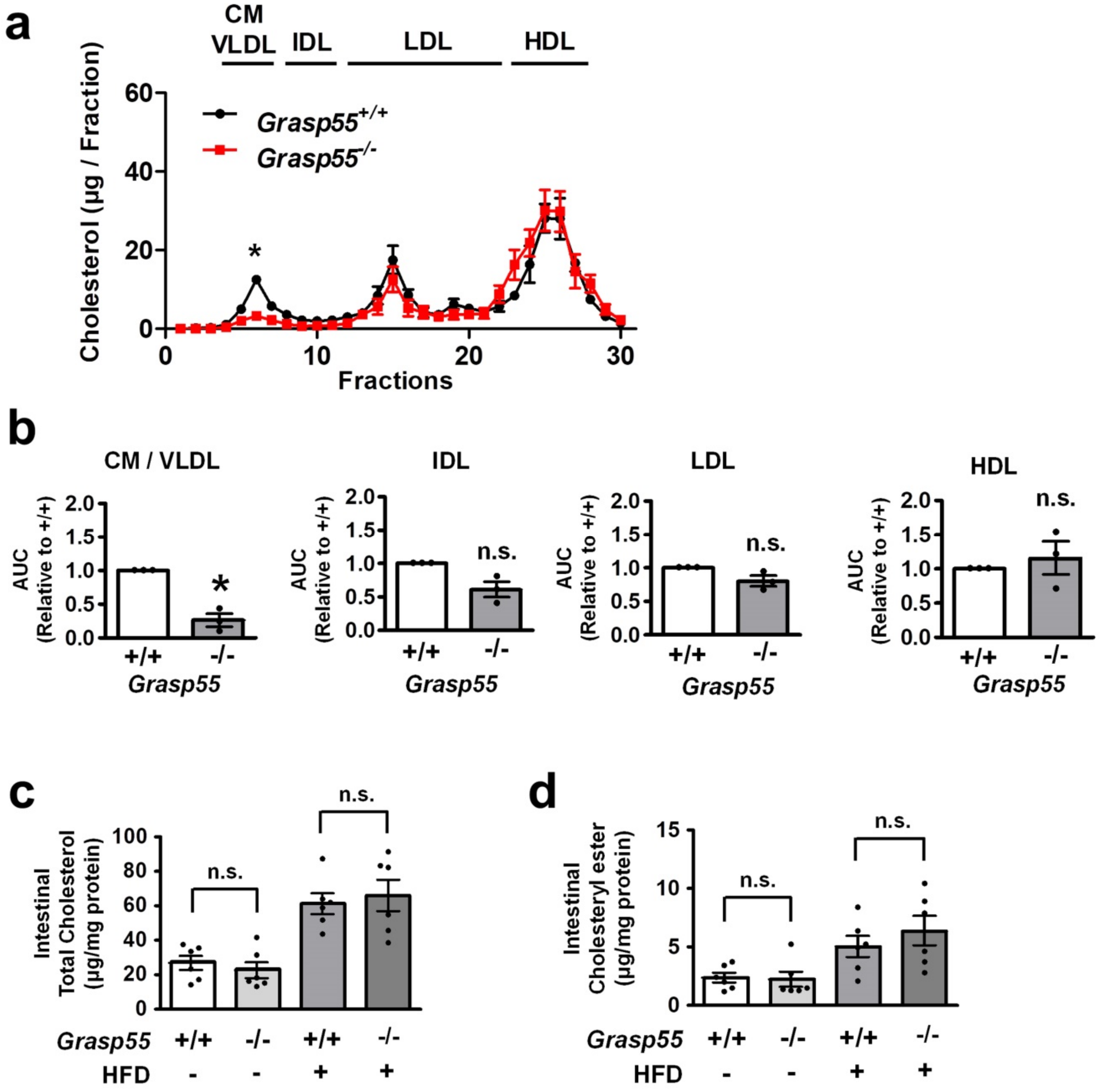

Supplementary Fig. 5 | Measurement of cholesterol in plasma fractions and intestines. a, **b,** Cholesterol content was determined in each blood plasma fraction after fast performance liquid chromatography of plasma lipids from *Grasp55*$^{+/+}$ and *Grasp55*$^{-/-}$ mice. Blood samples were taken 2 h after oral gavage with olive oil. The cholesterol content in each fraction is presented in (**a**). The area under curve (AUC) of plasma lipoprotein fractions was measured using ImageJ software and is summarized in (**b**). The data represent three independent experiments using pooled blood plasma from three to four mice. GRASP55 deletion reduces cholesterol contents in chylomicron (CM)/VLDL fractions. **c**, **d**, Intestinal cholesterol (**c**) and cholesteryl ester (**d**) levels were measured in the jejunum in 12-week-old *Grasp55*$^{+/+}$ and *Grasp55*$^{-/-}$ mice fasted for 16 h (HFD -), followed by high-fat diet feeding for 4 h (HFD +) (n = 6). Data are shown as mean ± SEM. n.s.: not significant, *$p < 0.05$. *P* values were calculated by paired (**b**) or unpaired (**c** and **d**) two-tailed Student's *t* tests. Source data are provided as a Source Data file.

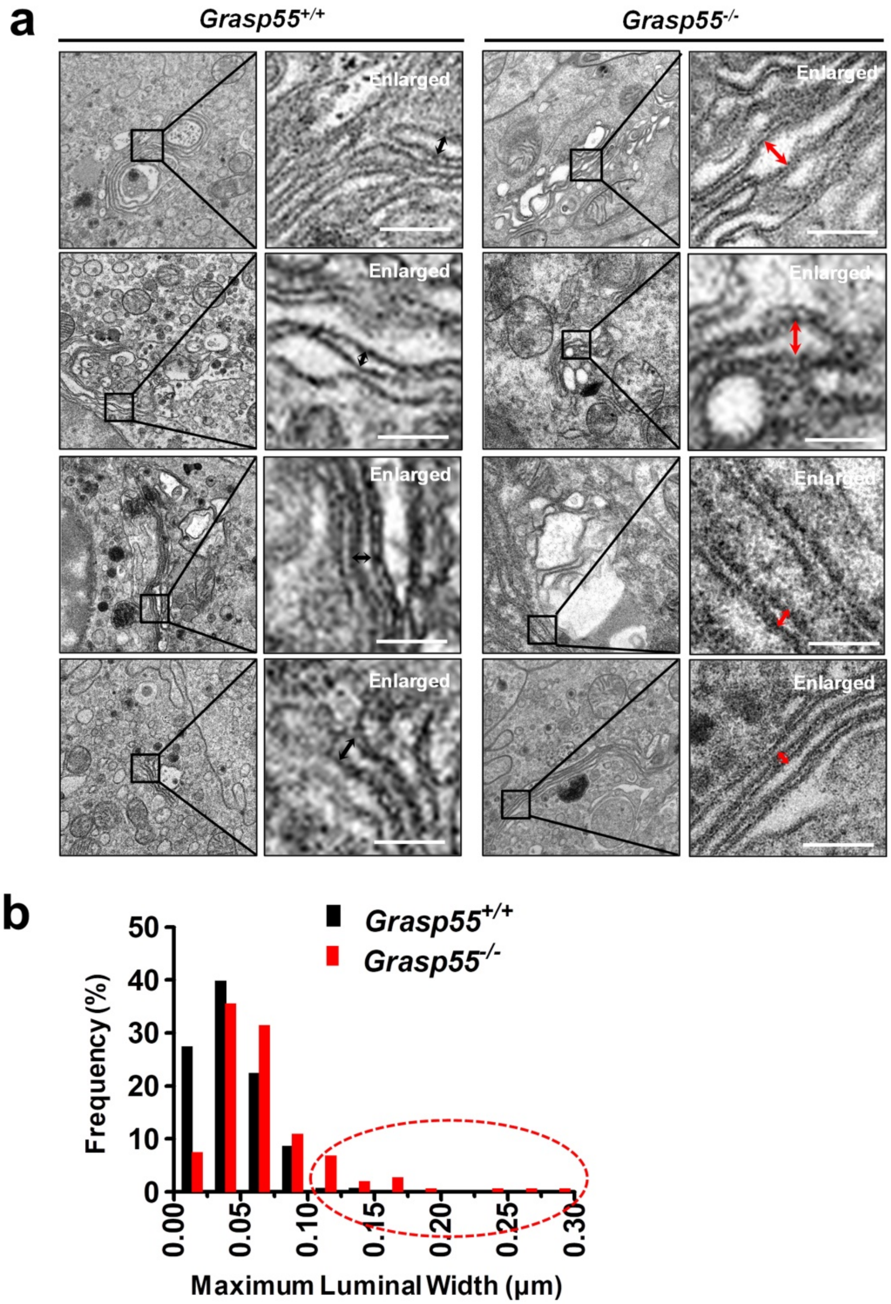

Supplementary Fig. 6 | The maximum luminal width of the Golgi cisternae is increased by GRASP55 deficiency. a, The maximum lumen widths of the Golgi cisternae were measured using EM images of mouse intestinal cells as detailed in Methods. Jejunum tissues of 12-week-old male mice were prepared 16 h after fasting. Bidirectional arrows indicate the maximum luminal width. **b**, Quantitative analyses of the maximum luminal width of the Golgi cisternae are summarized (*Grasp55*$^{+/+}$, n = 138 from five mice; *Grasp55*$^{-/-}$, n = 146 from five mice). GRASP55 deficiency did not cause Golgi unstacking. However, it appeared to increase the luminal width of some Golgi cisternae (>0.1 µm, dashed circle in **b**). On average, GRASP55 deficiency increased the maximum luminal width from 49.9 ± 2.2 nm to 58.1 ± 3.8 nm with a marginal statistical significance (p = 0.065, Student's t test). Scale bars: 200 nm. Source data are provided as a Source Data file.

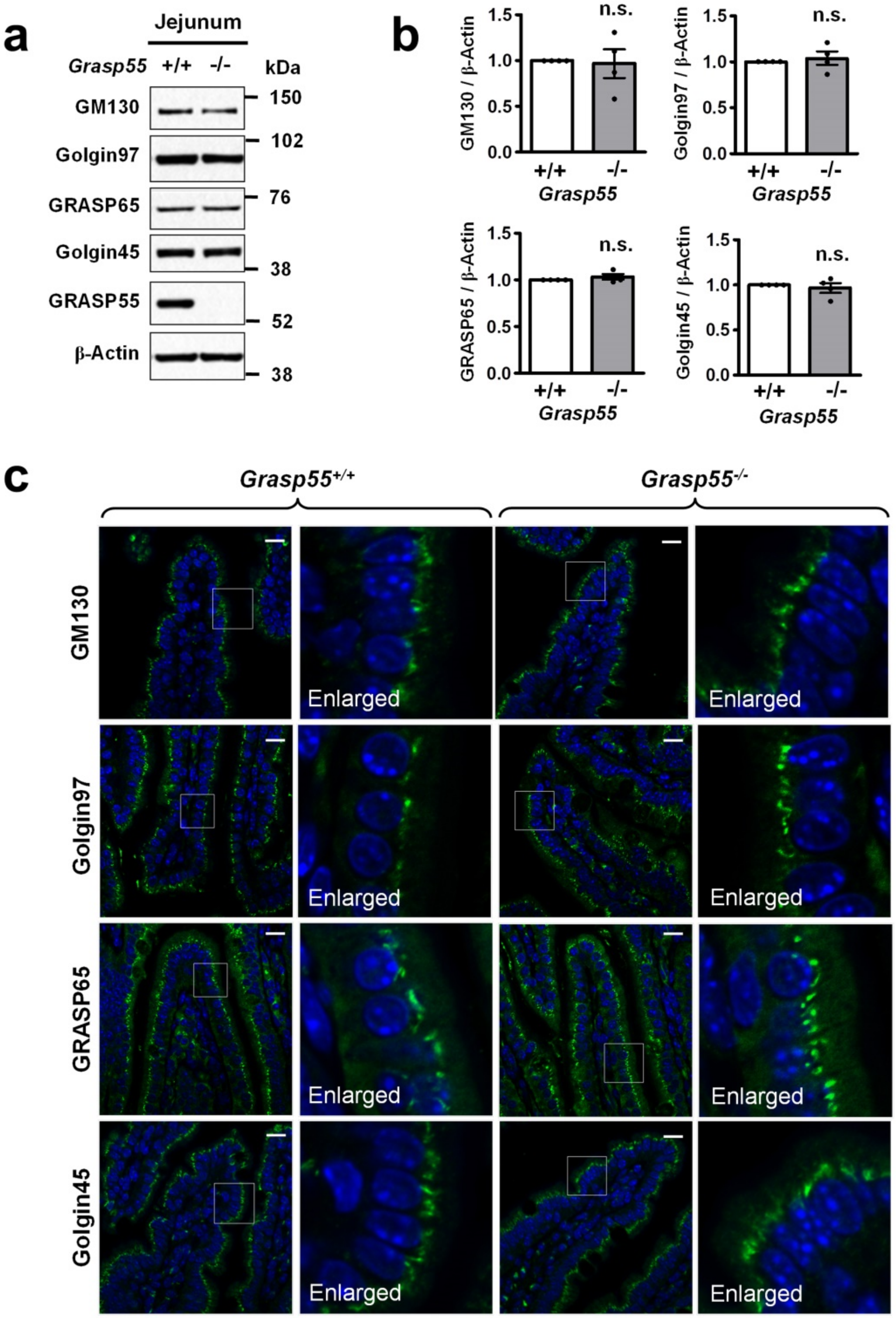

Supplementary Fig. 7 | Golgi cisternal adhesive proteins are not altered by GRASP55 deficiency in the mouse jejunum. **a**, **b**, Immunoblot analysis of GM130, Golgin97, GRASP65, and Golgin45. Representative immunoblots are shown in (**a**). Densitometric analyses are shown in (**b**) (n = 4 for each). β-actin was monitored as a cytosolic protein loading control. **c**, Immunohistochemistry was performed using jejunums of *Grasp55*$^{+/+}$ and *Grasp55*$^{-/-}$ mice. GRASP55 deletion did not alter the Golgi localization of GM130, Golgin97, GRASP65, and Golgin45 (green, apical side of perinuclear regions in the jejunal epithelia). Nuclei were counterstained with 4′,6-diamidino-2-phenylindole (blue). Three independent experiments showed similar results. Unprocessed blots are presented in Supplementary Fig. 22. Data are shown as mean ± SEM. n.s.: not significant. All p values were calculated by paired two-tailed Student's t tests. Scale bars: 20 μm. Source data are provided as a Source Data file.

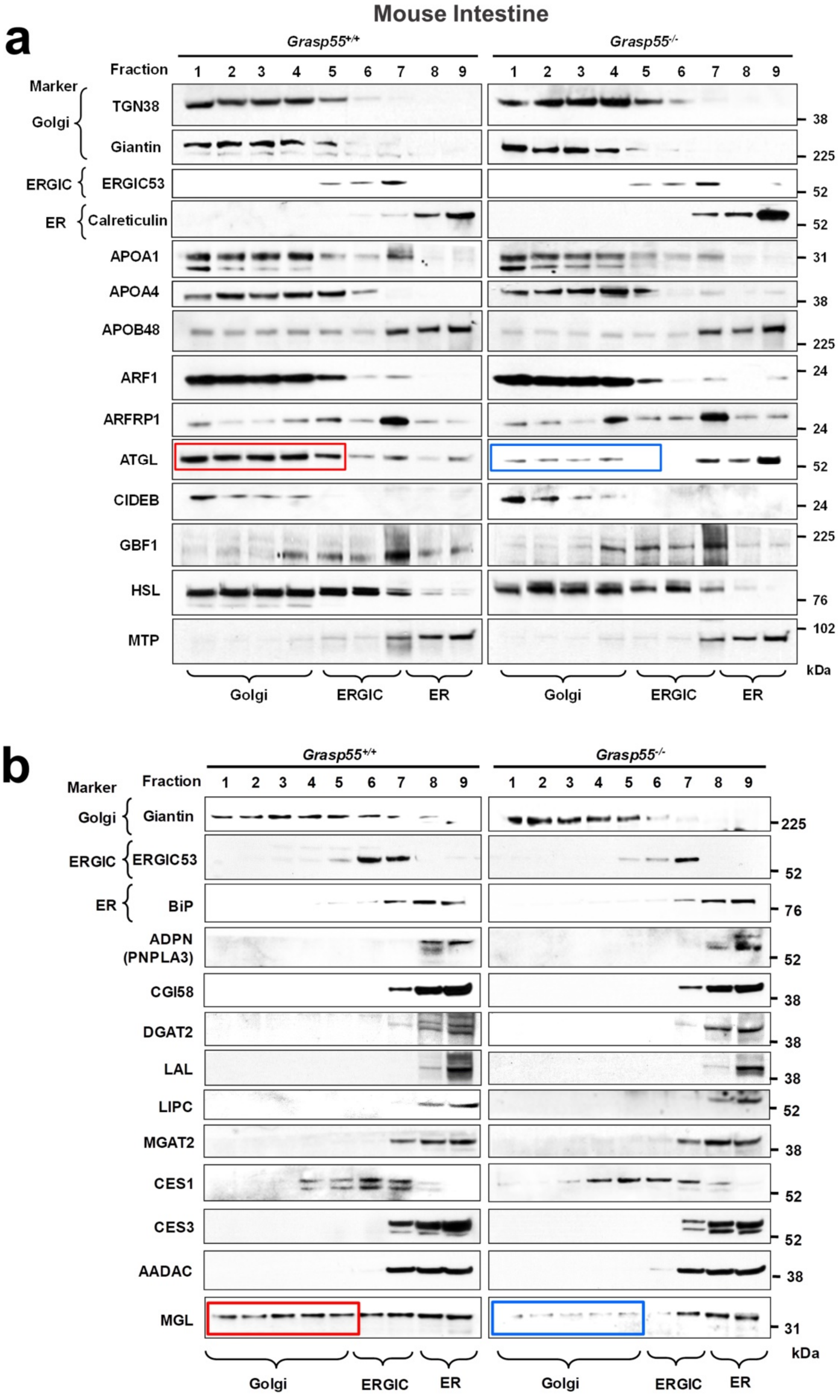

Supplementary Fig. 8 | See next page for caption.

Supplementary Fig. 8 | Subcellular localizations of ATGL and MGL are altered by GRASP55 deficiency in mouse intestines. Membrane fractionation analysis was performed using intestinal epithelial cells of *Grasp55*$^{+/+}$ and *Grasp55*$^{-/-}$ mice fed normal diet. Membrane fractions of jejunal epithelial cells were separated by density gradient ultracentrifugation using an OptiPrep gradient, followed by immunoblotting with the indicated antibodies. Organelle markers used are as follows: Giantin (for the Golgi), ERGIC53 (for the ER–Golgi intermediate compartment), Calreticulin (**a**, for the ER), and BiP (**b**, for the ER). The Golgi localizations of ATGL (**a**) and MGL (**b**) were markedly reduced by GRASP55 deficiency in mouse intestinal cells (red and blue boxes). Three independent experiments were performed and showed similar results. AADAC, arylacetamide deacetylase; ADPN, Adiponutrin (PNPLA3, patatin-like phospholipase domain-containing protein 3); APOA1, apolipoprotein A1; APOA4, apolipoprotein A4; APOB48, apolipoprotein B48; ARF1, ADP-ribosylation factor 1; ARFRP1, ADP-ribosylation factor-related protein 1; ATGL, adipose triglyceride lipase; CES1, carboxylesterase 1; CES3, carboxylesterase 3; CIDEB, cell death-inducing DFFA-like effector B; CGI58, comparative gene identification-58; DGAT2, diglyceride acyltransferase 2; GBF1, Golgi-specific brefeldin A-resistance guanine nucleotide exchange factor 1; HSL, hormone-sensitive lipase; LAL, lysosomal acid lipase; LIPC, hepatic triglyceride lipase; MGAT2, monoacylglycerol acyltransferase-2; MGL, monoglyceride lipase; MTP, microsomal triglyceride transfer protein. Unprocessed blots can be found in Supplementary Fig. 22. Source data are provided as a Source Data file.

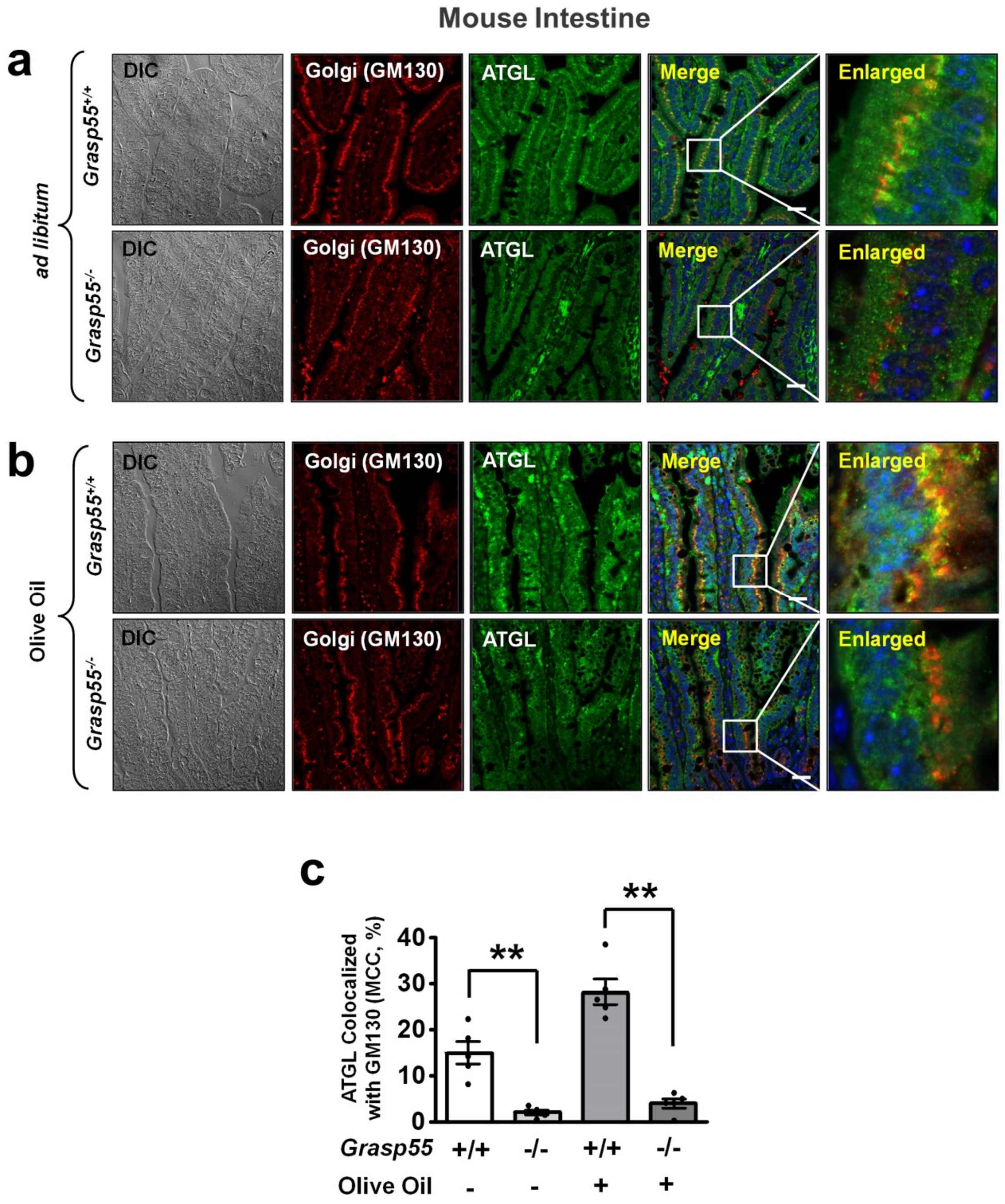

Supplementary Fig. 9 | GRASP55 deficiency reduces the Golgi localization of ATGL. Immunohistological images of ATGL and the Golgi marker protein GM130 in intestinal epithelia. Jejunum tissues were prepared from *Grasp55*$^{+/+}$ and *Grasp55*$^{-/-}$ mice with free access to food (**a**) or 4 h after olive oil bolus (**b**, olive oil, 10 µl/g of body weight). The results of multiple experiments (n = 5) are summarized in (**c**). MCC, Manders' colocalization coefficient. Scale bars: 20 µm. Data are shown as mean ± SEM. $^{**}p < 0.01$. All p values were calculated by unpaired two-tailed Student's t tests. Source data are provided as a Source Data file.

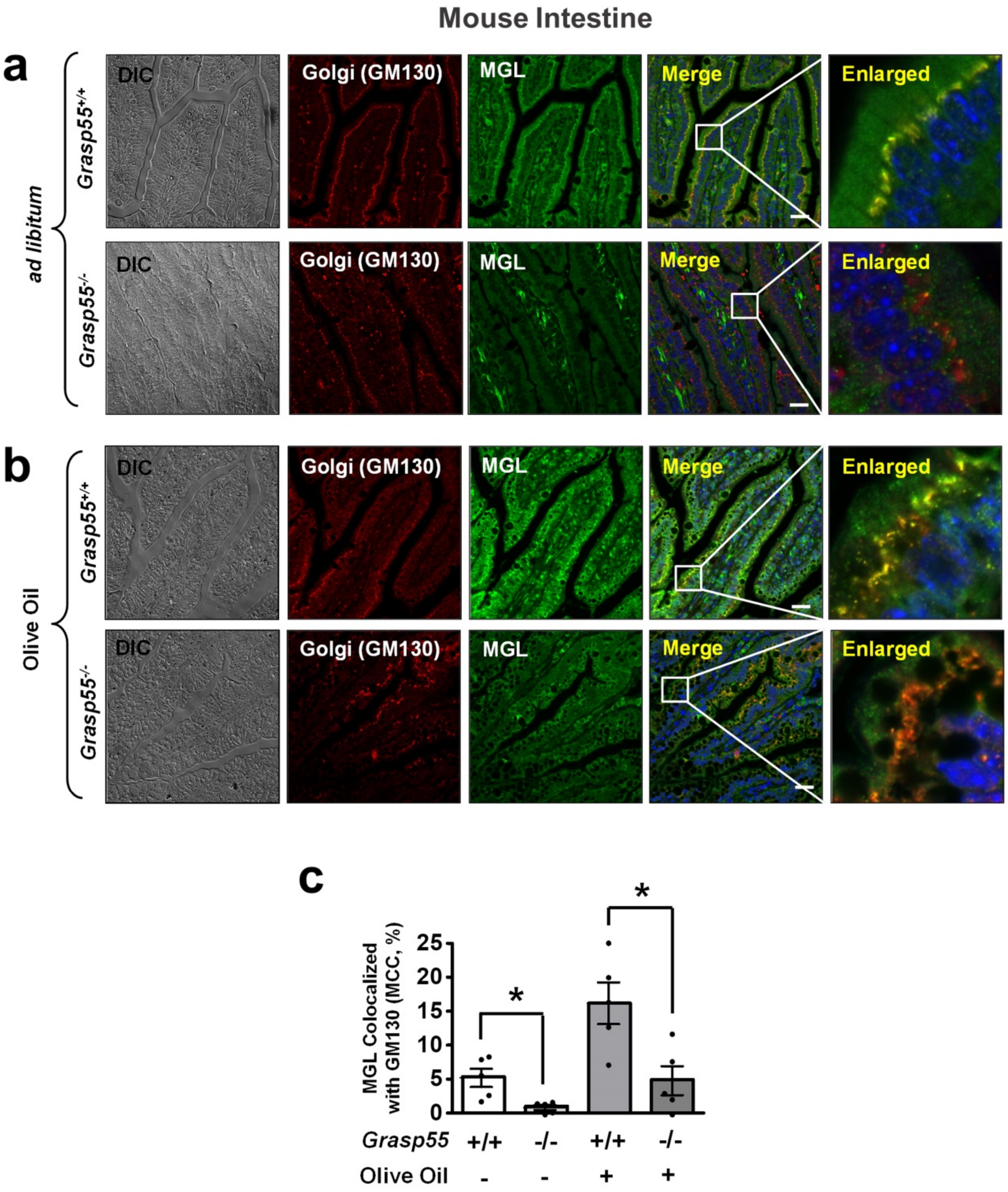

Supplementary Fig. 10 | GRASP55 deficiency reduces the Golgi localization of MGL. Immunohistological images of MGL and the Golgi marker protein GM130 in intestinal epithelia. Jejunum tissues were prepared from *Grasp55*$^{+/+}$ and *Grasp55*$^{-/-}$ mice with free access to food (**a**) or 4 h after olive oil bolus (**b**, olive oil, 10 µl/g of body weight). The results of multiple experiments (n = 5) are summarized (**c**). MCC, Manders' colocalization coefficient. Scale bars: 20 µm. Data are shown as mean ± SEM. *$p < 0.05$. All p values were calculated by unpaired two-tailed Student's t tests. Source data are provided as a Source Data file.

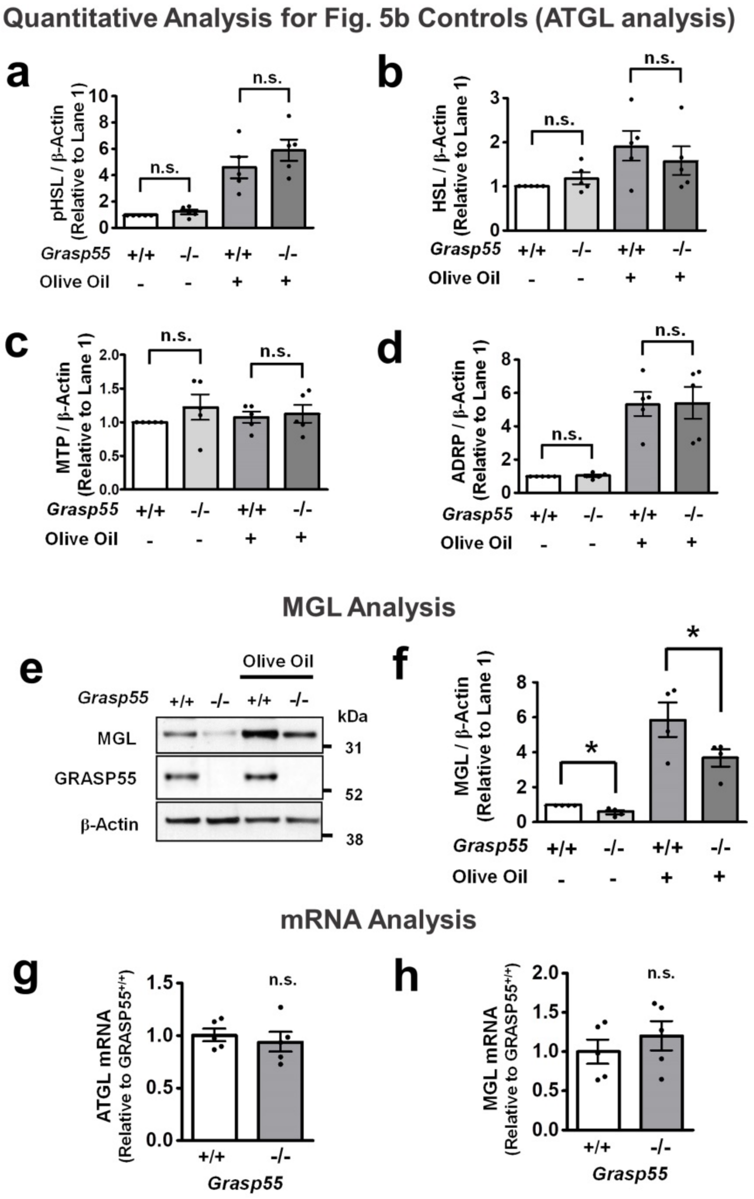

Supplementary Fig. 11 | *Grasp55* deficiency reduces cellular protein levels of MGL, but does not affect those of pHSL, HSL, MTP, or ADRP in mouse intestines. a–d, Densitometric analyses of pHSL (**a**), HSL (**b**), MTP (**c**), and ADRP (**d**) are presented (n = 5 each). Representative immunoblots are shown in Fig. 5b. The levels of pHSL, HSL, and MTP were not altered by GRASP55 depletion. **e, f,** The protein expression of MGL was analyzed by immunoblotting. Representative immunoblots are shown in (**e**) and densitometric analysis of multiple experiments is shown in (**f**, n = 4). The level of β-actin was monitored as a cytosolic protein loading control. **g, h,** Quantitative PCR analysis of ATGL (**g**, n = 5) and MGL (**h**, n = 5) mRNAs. GRASP55 depletion reduces cytosolic MGL levels without affecting its mRNA levels. Unprocessed blots can be found in Supplementary Fig. 22. HSL, hormone-sensitive lipase; MGL, monoglyceride lipase; MTP, microsomal triglyceride transfer protein. Data are shown as mean ± SEM. n.s.: not significant, *$p < 0.05$. All p values were calculated by unpaired two-tailed Student's t tests. Source data are provided as a Source Data file.

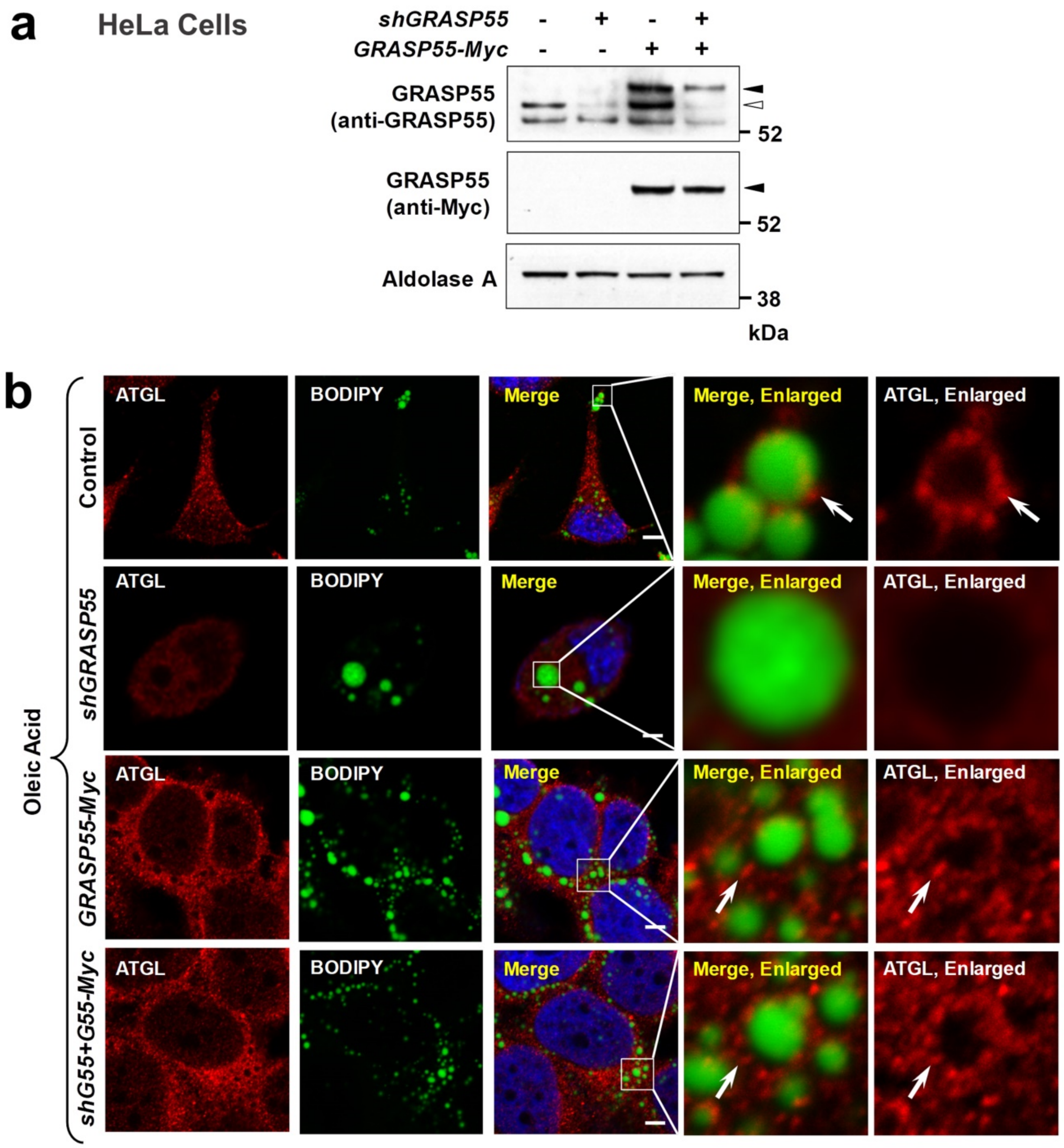

Supplementary Fig. 12 | GRASP55 depletion induces failure in the LD-targeting of ATGL in HeLa Cells. a, The expression levels of GRASP55 proteins were examined in HeLa cells, in which GRASP55 was depleted (*shGRASP55*, *shG55,* open arrowhead) and/or exogenously supplemented (*GRASP55-Myc*, *G55-Myc,* filled arrowhead). Aldolase A was used as a cytosolic protein loading control. **b**, Presence of ATGL in LD surface was examined by co-staining with anti-ATGL antibodies and BODIPY 493/503 (an LD marker) in HeLa cells following treatment with 400 µM oleic acid for 4 h. Arrows indicate the location of ATGL targeted to the peripheral regions of LDs. Three independent experiments showed similar results. Unprocessed blots are presented in Supplementary Fig. 22. Scale bars: 10 µm. Source data are provided as a Source Data file.

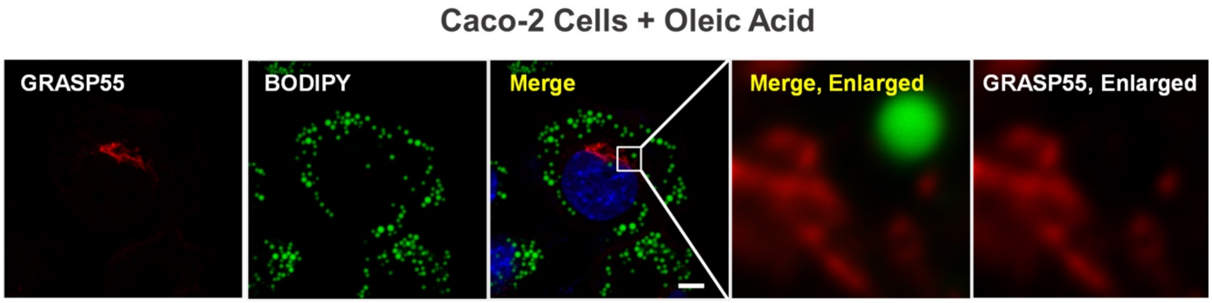

Supplementary Fig. 13 | GRASP55 does not localize on LDs. Localization of GRASP55 was examined by co-staining with anti-GRASP55 antibodies and BODIPY 493/503 (an LD marker) in Caco-2 cells following treatment with 400 μM oleic acid for 16 h. Three independent experiments showed similar results. Scale bars: 10 μm.

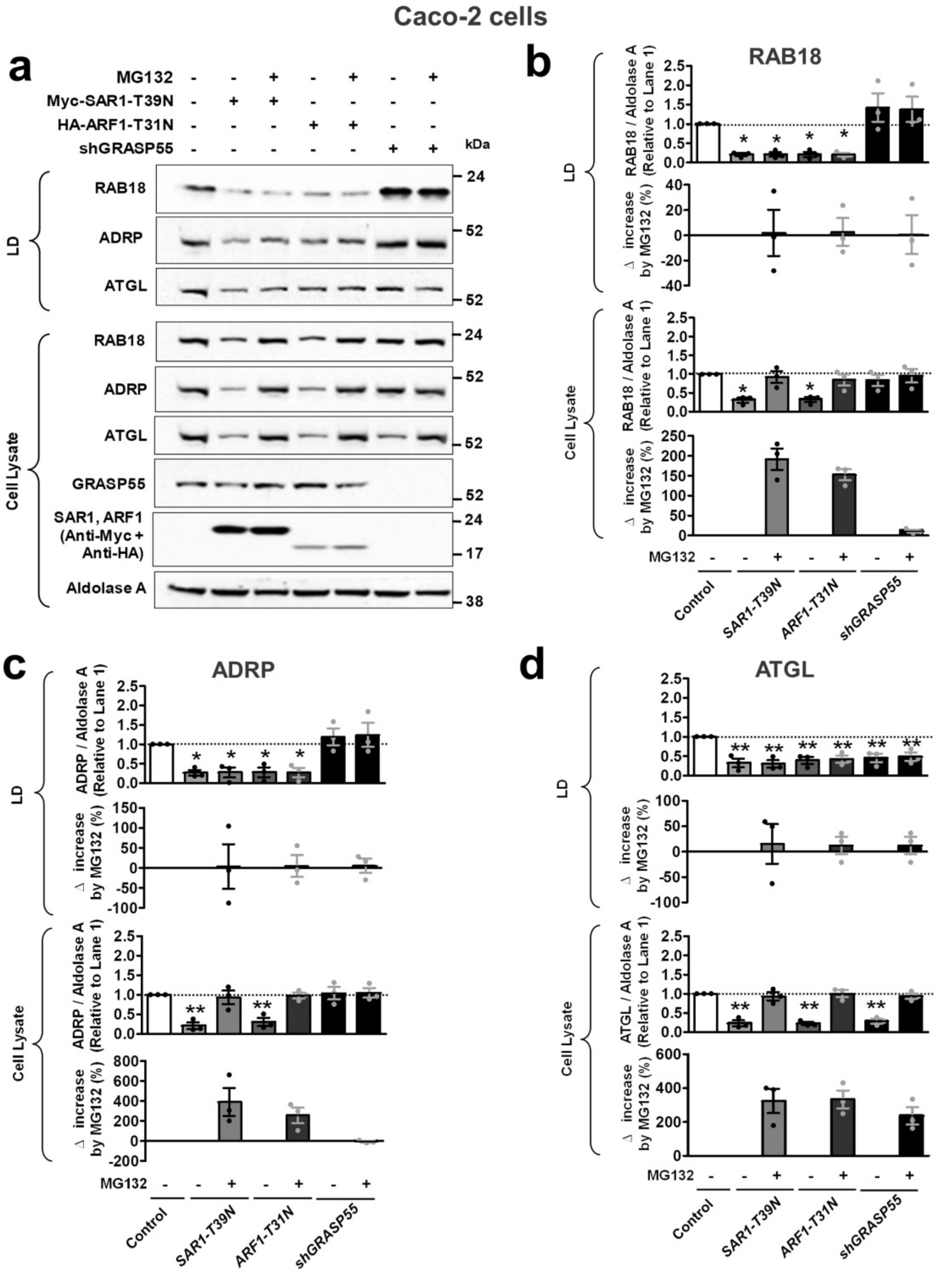

Supplementary Fig. 14 | Decreased ATGL levels in LDs by ER-to-Golgi blockade are not reversed by proteasome inhibition. Immunoblot analyses of ATGL levels in LDs and cell lysates of Caco-2 cells were performed in the absence or presence of the proteasomal inhibitor MG132 (0.5 μM, 16 h). The ER-to-Golgi trafficking was blocked by dominant-inhibitory forms of SAR1 (SAR1-T39N) and ARF1 (ARF1-T31N). For the induction of ATGL protein expression, cells were treated with 400 μM oleic acid for 16 h. Representative immunoblots of LDs and cytosols are shown in (**a**). In panels (**b–d**), the results of multiple experiments of RAB18, ADRP, and ATGL, respectively, are summarized (n = 3 each). Aldolase A was used as a cytosolic protein loading control. MG132 does not rescue the decreased RAB18, ADRP, or ATGL levels induced by SAR1-T39N and ARF1-T31N. Unprocessed blots can be found in Supplementary Fig. 22. Data are shown as mean ± SEM. *$p < 0.05$. **$p < 0.01$. All p values were calculated by ANOVA followed by Tukey's multiple comparison tests. Source data are provided as a Source Data file.

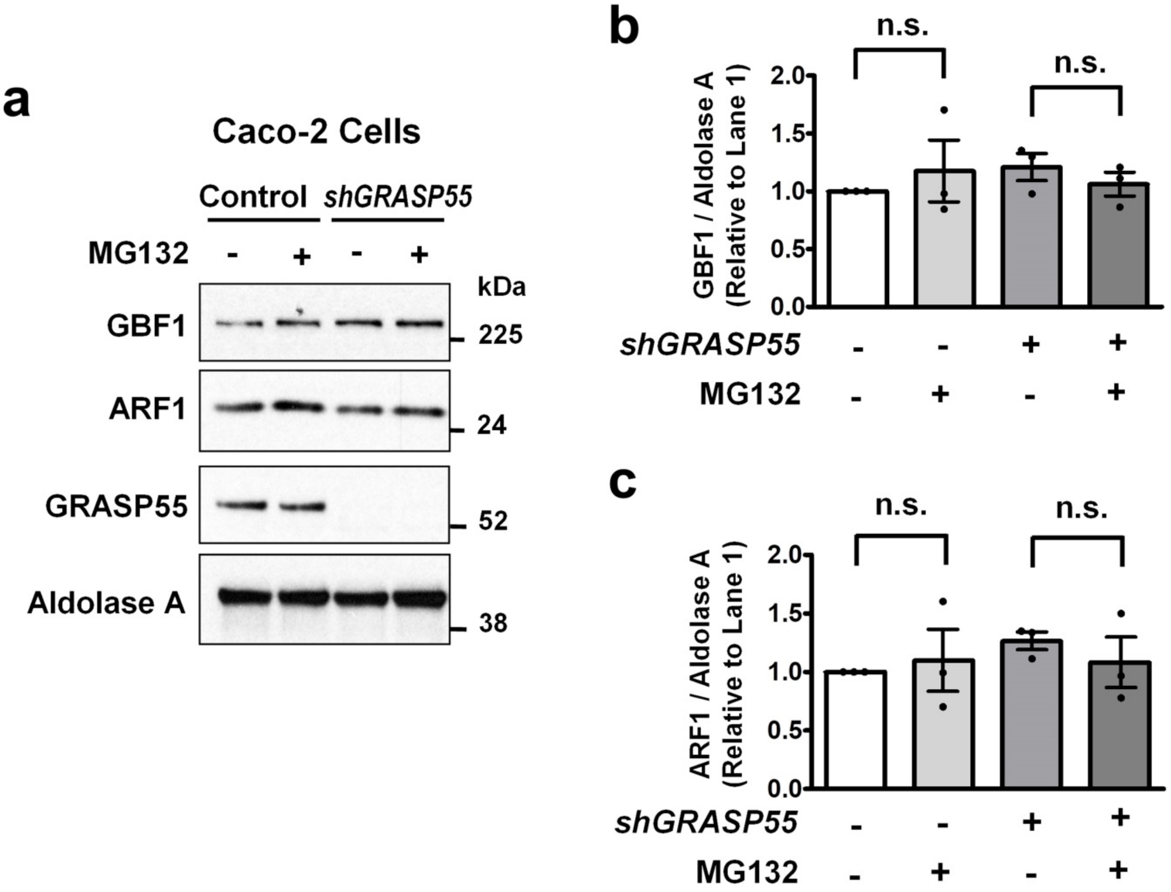

Supplementary Fig. 15 | GRASP55 does not alter the protein stability of GBF1 and ARF1 in Caco-2 cells. Immunoblot analysis of GBF1 and ARF1 levels were performed in control and GRASP55-depleted Caco-2 cells, stably transfected with shRNA against GRASP55 (*shGRASP55*). Some cells were incubated with the proteasomal inhibitor MG132 (0.5 µM, 16 h). Representative immunoblots are shown in (**a**). Densitometric analyses of GBF1 and ARF1 are shown in (**b**) and (**c**), respectively (n = 3 for each). GBF1 and ARF1 levels were not altered by GRASP55 depletion or by treatment with MG132 (compare with the results obtained for ATGL, shown in Supplementary Fig. 13). Unprocessed blots are presented in Supplementary Fig. 22. Data are shown as mean ± SEM. n.s.: not significant. All *p* values were calculated by unpaired two-tailed Student's *t* tests. Source data are provided as a Source Data file.

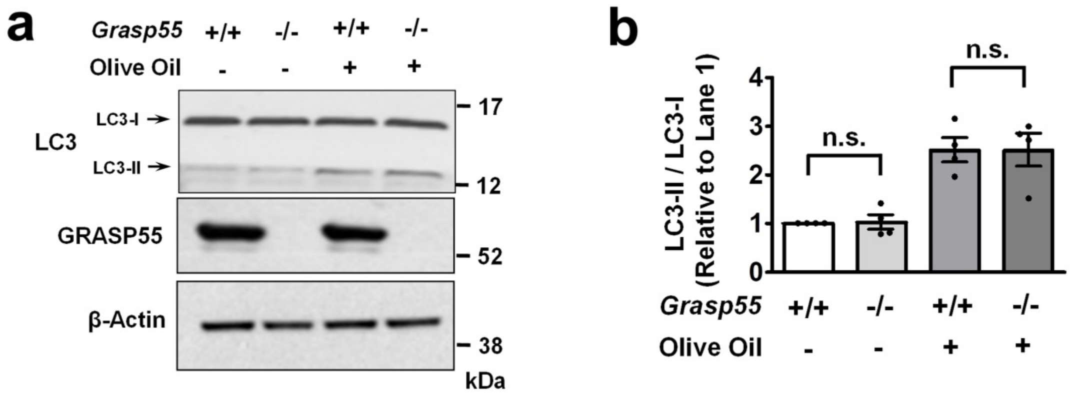

Supplementary Fig. 16 | Autophagic flux is not altered by *Grasp55* deletion in the mouse jejunum. To analyze the effects of GRASP55 deficiency on autophagy, conversion of microtubule-associated protein light chain 3 (LC3) into its phosphatidylethanolamine-conjugated form (LC3-II) as a parameter of autophagic flux was examined. The jejunum samples were taken from ad libitum-fed mice. Some samples were taken 2 h after olive oil feeding (10 μl/g body weight). Under both conditions, deletion of *Grasp55* did not affect LC3-II conversion. Representative immunoblots (**a**) and the results of densitometric analyses (**b**) (n = 4) are shown. Unprocessed blots are presented in Supplementary Fig. 22. Data are shown as mean ± SEM. n.s.: not significant. All *p* values were calculated by unpaired two-tailed Student's *t* tests. Source data are provided as a Source Data file.

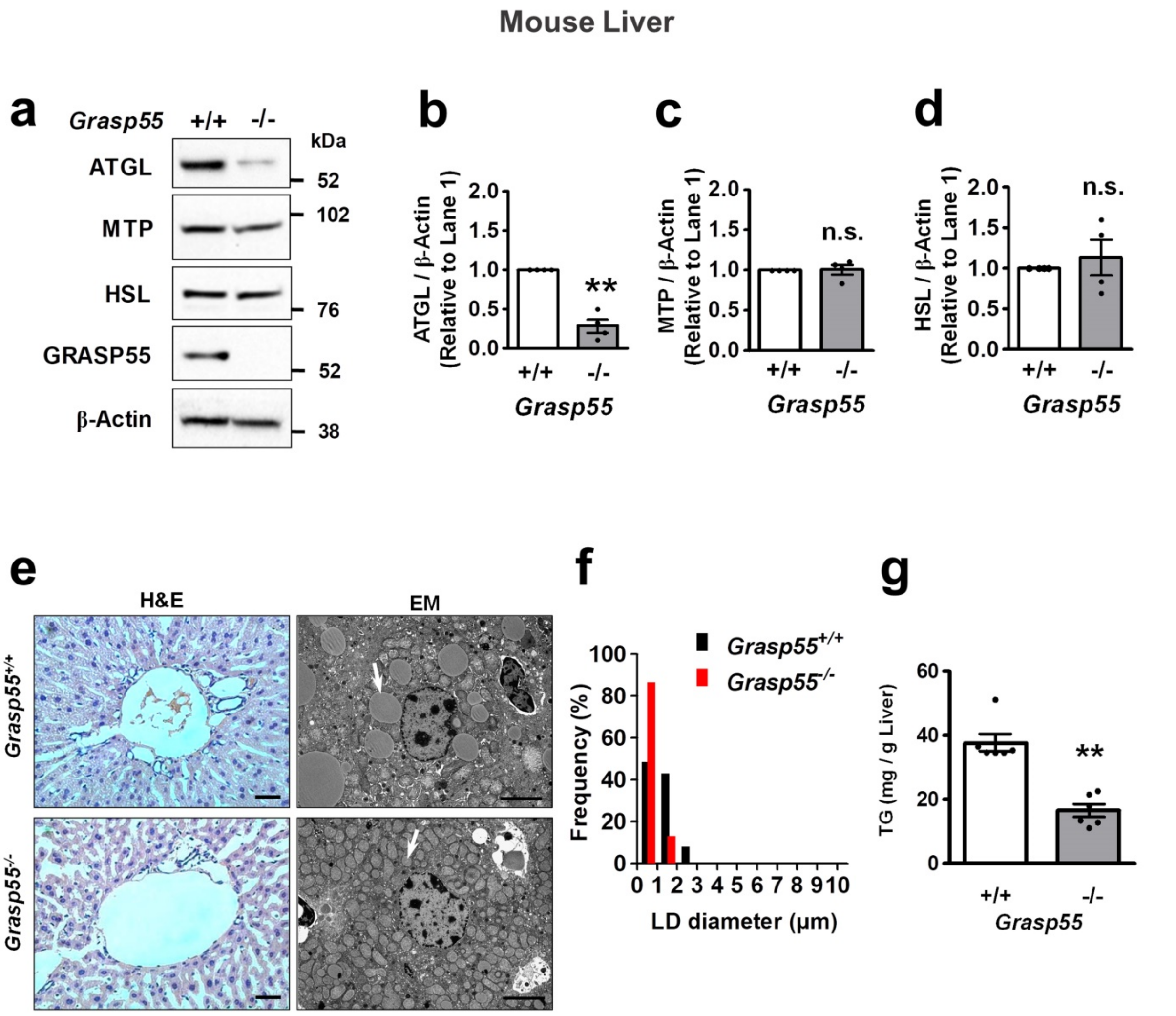

Supplementary Fig. 17 | GRASP55 deficiency decreases the cellular levels of ATGL and triglyceride contents in mouse hepatocytes. a-d, Immunoblot analysis of ATGL, MTP, and HSL in the mouse liver. Liver tissues were prepared from 12-week-old *Grasp55*$^{+/+}$ and *Grasp55*$^{-/-}$ mice fed on the normal diet. A representative immunoblot is shown in (**a**), and the results of multiple experiments (n = 4) are summarized in (**b-d**). The amount of β-actin was monitored as a cytosolic protein loading control. **e**, **f,** Morphological analysis of mouse livers fasted for 16 h or after olive oil bolus (olive oil, 10 μl/g of body weight) by H&E staining and electro-microscopy (EM). White arrows indicate the cytosolic LDs. Note that LD size is reduced in *Grasp55*$^{-/-}$ mice. Quantitative analyses of LD diameters are summarized in (**f,** *Grasp55*$^{+/+}$, n = 296 from five mice; *Grasp55*$^{-/-}$, n = 150 from five mice). GRASP55 deficiency reduced the LD size from 1.23 ± 0.04 μm to 0.73 ± 0.03 μm ($p < 0.01$). **g**, The amount of triglyceride (TG) per g liver was measured (n = 6). Unprocessed blots can be found in Supplementary Fig. 22. Data are shown as mean ± SEM. Scale bars: 50 μm (H&E) and 5 μm (EM). n.s.: not significant, ***p* < 0.01. *P* values were calculated by paired (**b**, **c** and **d**) or unpaired (**g**) two-tailed Student's *t* tests. Source data are provided as a Source Data file.

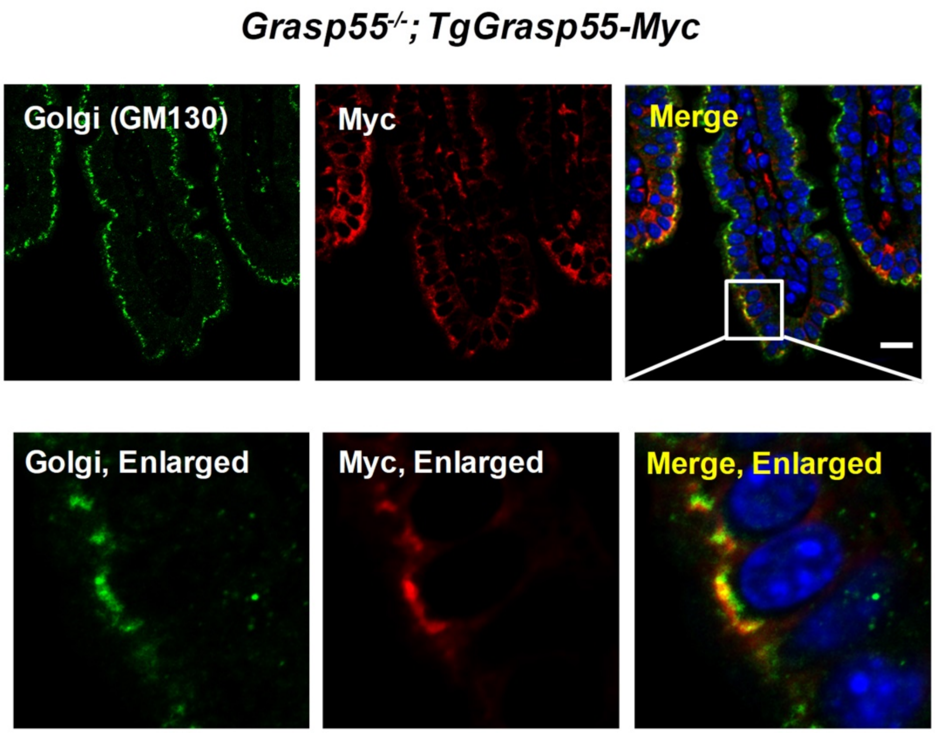

Supplementary Fig. 18 | Exogenous GRASP55 localizes on the Golgi in the jejunal epithelia of *TgGRASP55* mice. Immunohistological images of Golgi marker protein GM130 (green) and transgenic GRASP55 (GRASP55-Myc, red) in the intestinal epithelia were taken. Jejunum tissues were prepared from *Grasp55*$^{-/-}$*;TgGRASP55-Myc* mice. GM130 and GRASP55-Myc highly colocalized in the typical Golgi area (apical side of perinuclear regions) in the jejunal epithelia. Three independent experiments showed similar results. Scale bars: 20 μm.

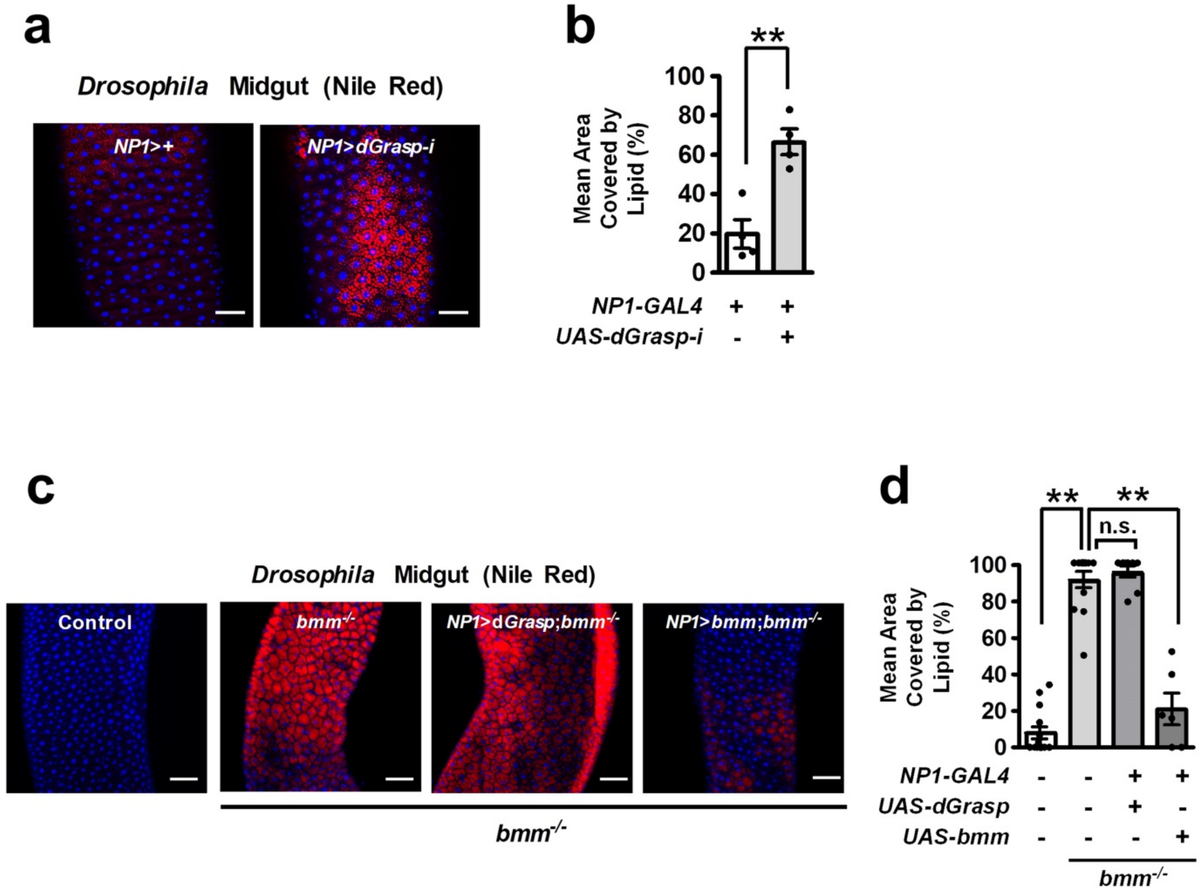

Supplementary Fig. 19 | Intestinal lipid accumulation in *NP1>dGrasp-i* and *bmm*$^{-/-}$ flies. Confocal microscopic images of lipid accumulation in the fly guts of indicated genotypes. Lipids were visualized by staining with Nile Red (red) and nuclei were counterstained with TO-PRO-3 (blue). **a**, **b** Lipid accumulation in the guts of enterocyte-specific *dGrasp*-depleted flies (*NP1>dGrasp-i*). Representative images of the posterior midguts are shown in (**a**), and quantitative analyses of intestinal lipid accumulation in the control (*NP1>+*) and *dGrasp*-depleted flies are shown in (**b**, n = 4). **c**, **d** Intestinal lipid accumulation in the *bmm*-deleted (*bmm*$^{-/-}$) flies. Representative images of the posterior midguts are shown in (**c**), and quantitative analyses of intestinal lipid accumulation in the indicated genotypes of the flies are shown in (**d**, n = 6–14). In contrast to enterocyte-specific *bmm* supplementation (*NP1>bmm,* 4th lane), *dGrasp* supplementation (*NP1>dGrasp,* 3rd lane) did not reduce lipid accumulation in the posterior midguts in *bmm*$^{-/-}$ flies. Data are shown as mean ± SEM. n.s.: not significant, $^{**}p < 0.01$. *P* values were calculated by unpaired two-tailed Student's *t* test (**b**) or ANOVA followed by Tukey's multiple comparison tests (**d**). Scale bars: 50 µm. Source data are provided as a Source Data file.

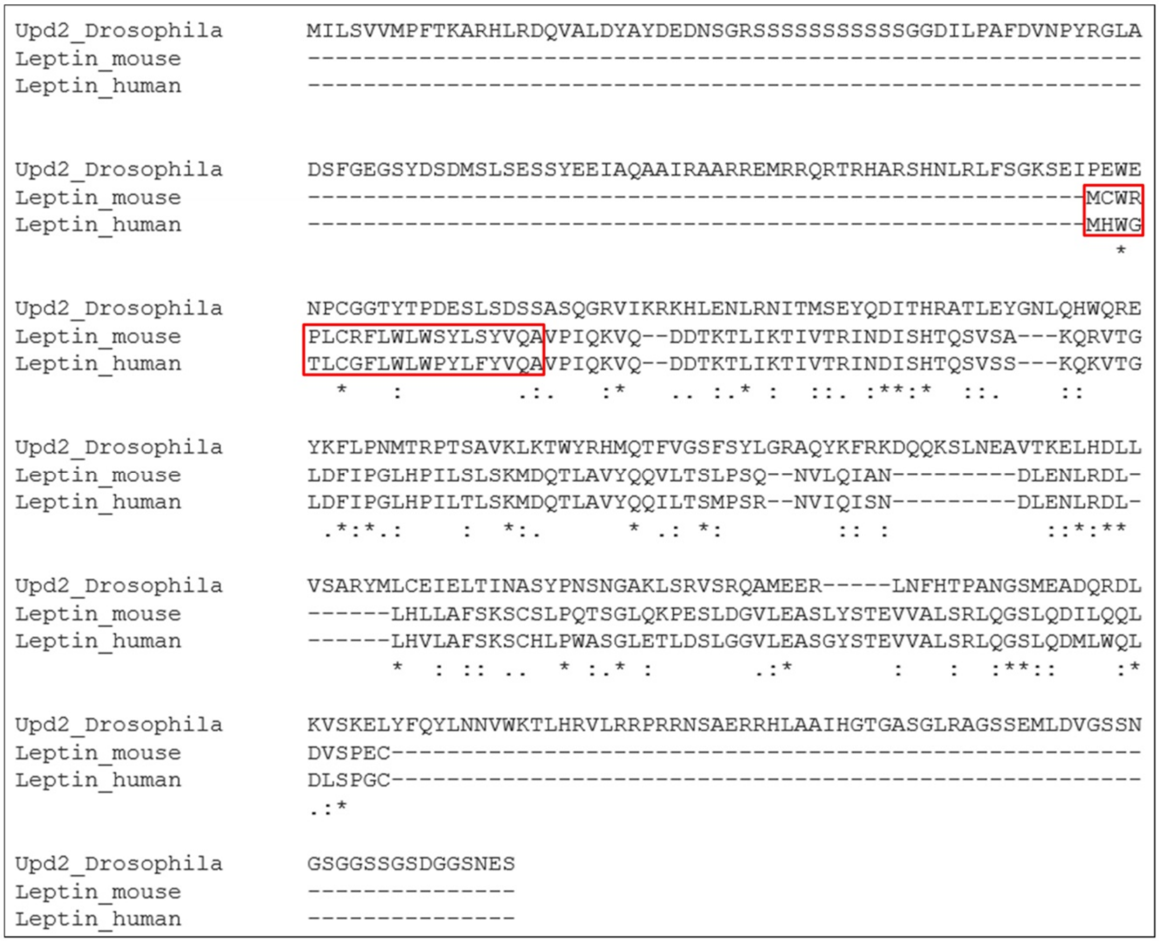

Supplementary Fig. 20 | Upd2 does not have the signal peptide sequence. The amino acid sequences of Upd2, *Drosophila* homolog of mammalian leptin, and mammalian leptins were aligned. The red box indicates the signal peptide sequence present in mouse and human leptins.

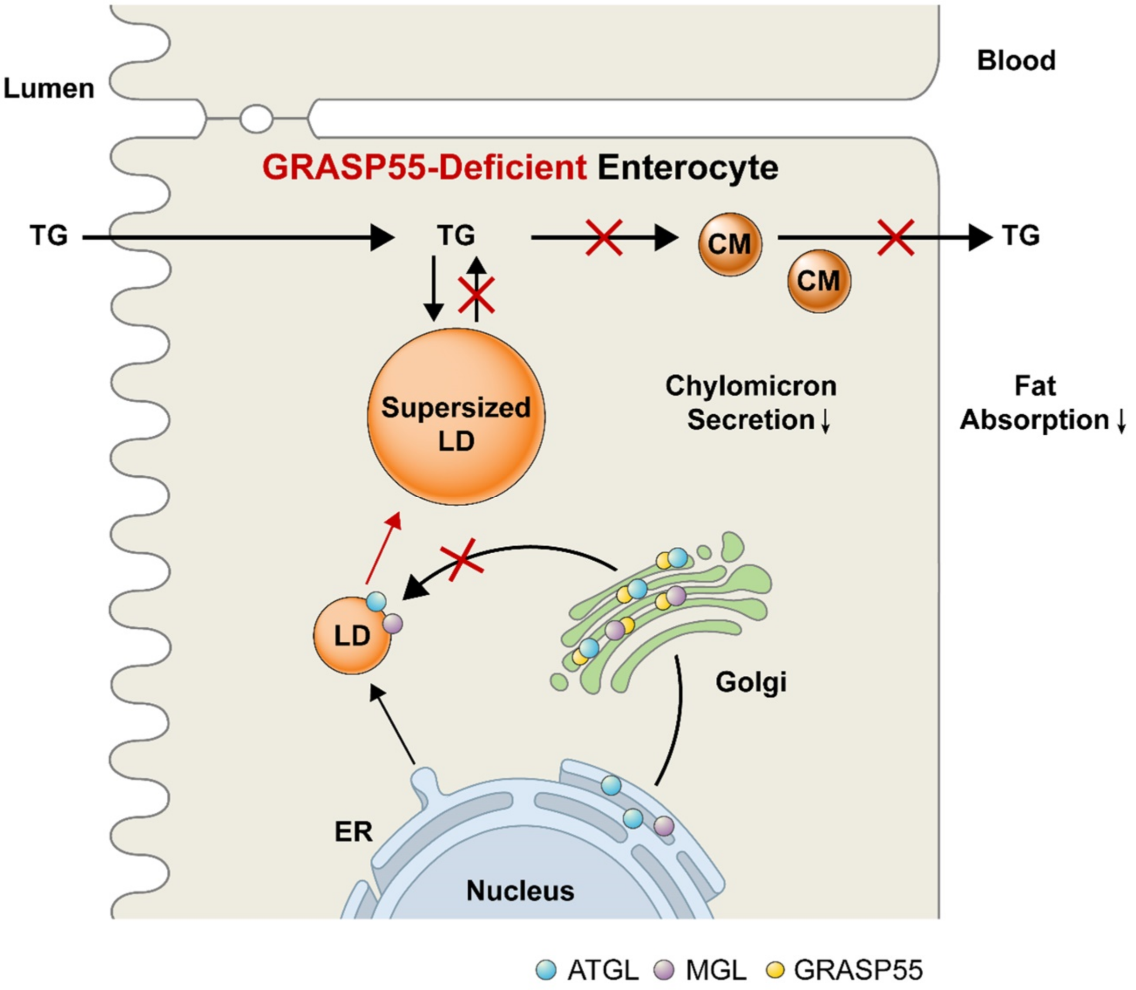

Supplementary Fig. 21 | Role of GRASP55 in intestinal fat absorption. Some LD-associated proteins, such as ATGL and MGL, reach their final LD destination via a Golgi- and GRASP55-dependent route. Defects in this process induce supersized LDs and reduce lipidation of chylomicrons in enterocytes upon exogenous lipid challenge, which eventually evokes reduced fat absorption.

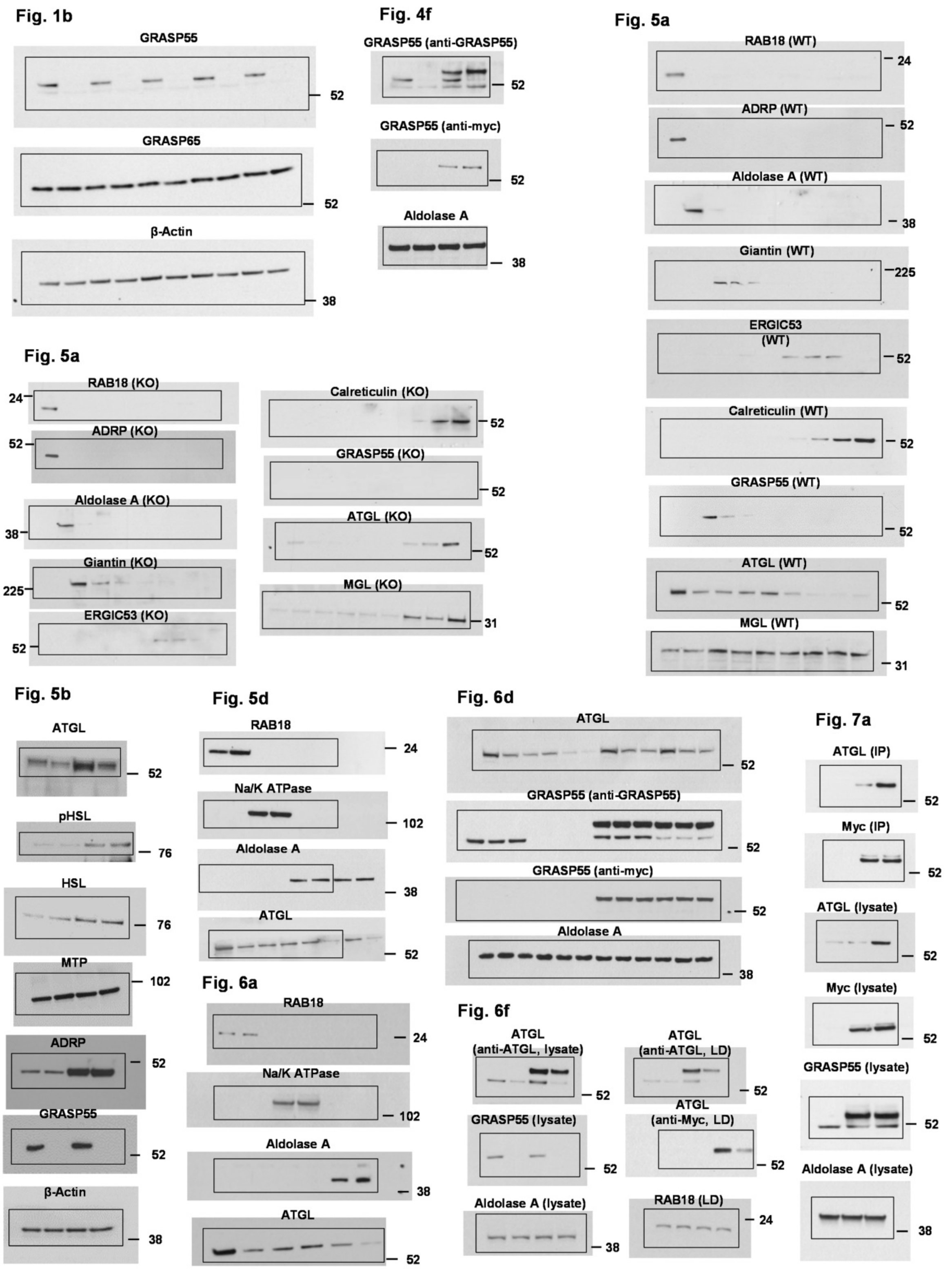

Supplementary Fig. 22 | Unprocessed images of blots in Figures 1–9 and Supplementary Figures 3–17.

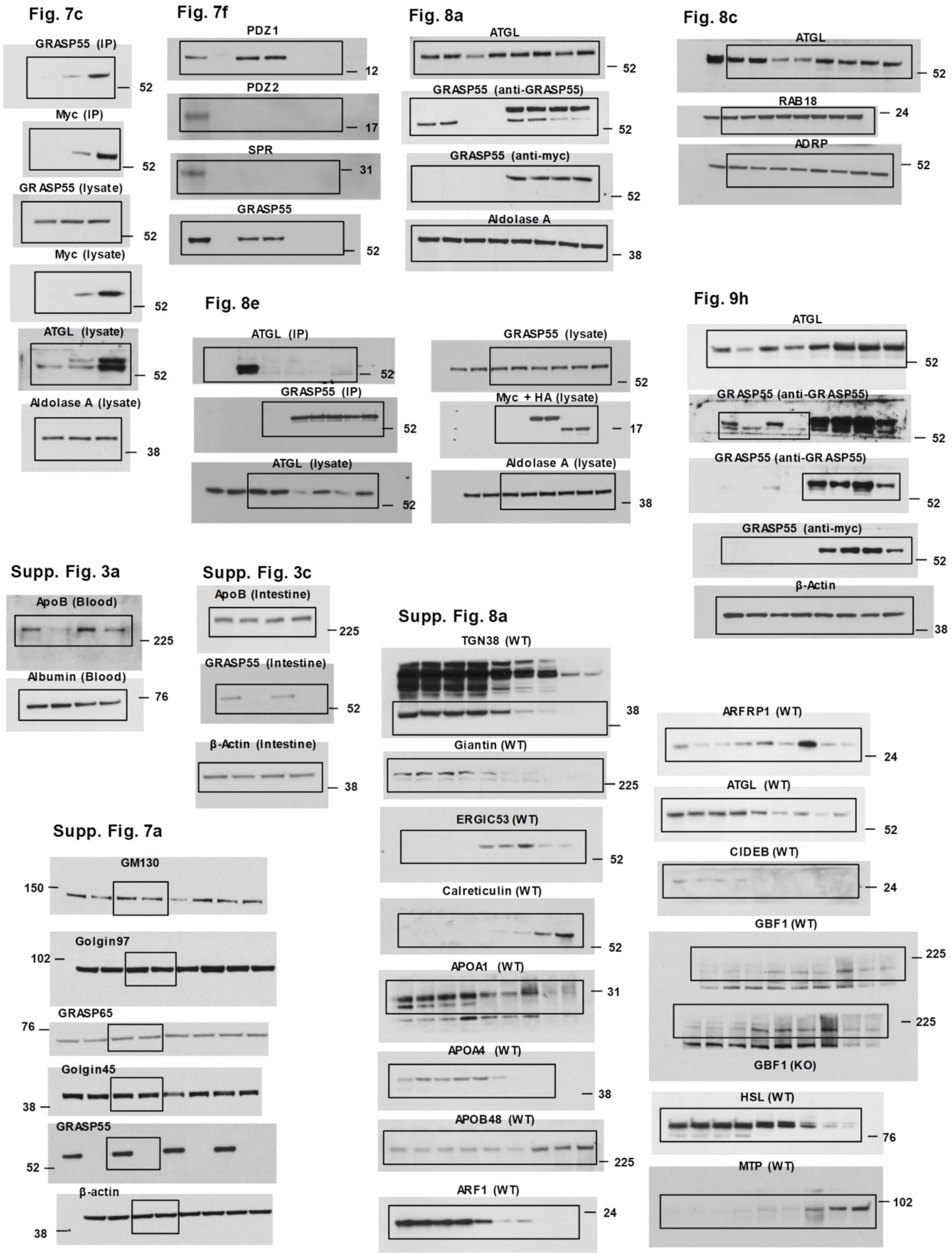

Supplementary Fig. 22 | Unprocessed images (continued)

Supp. Fig. 8a

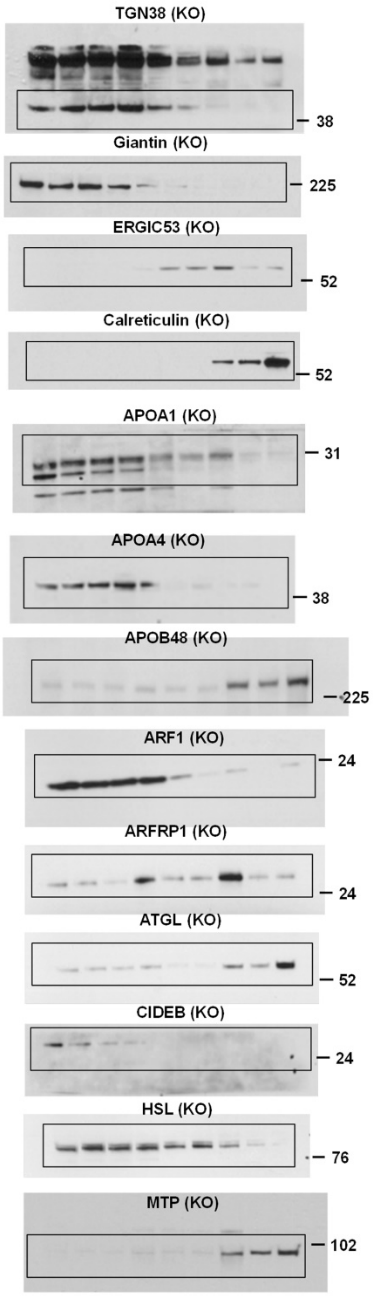

Supp. Fig. 8b

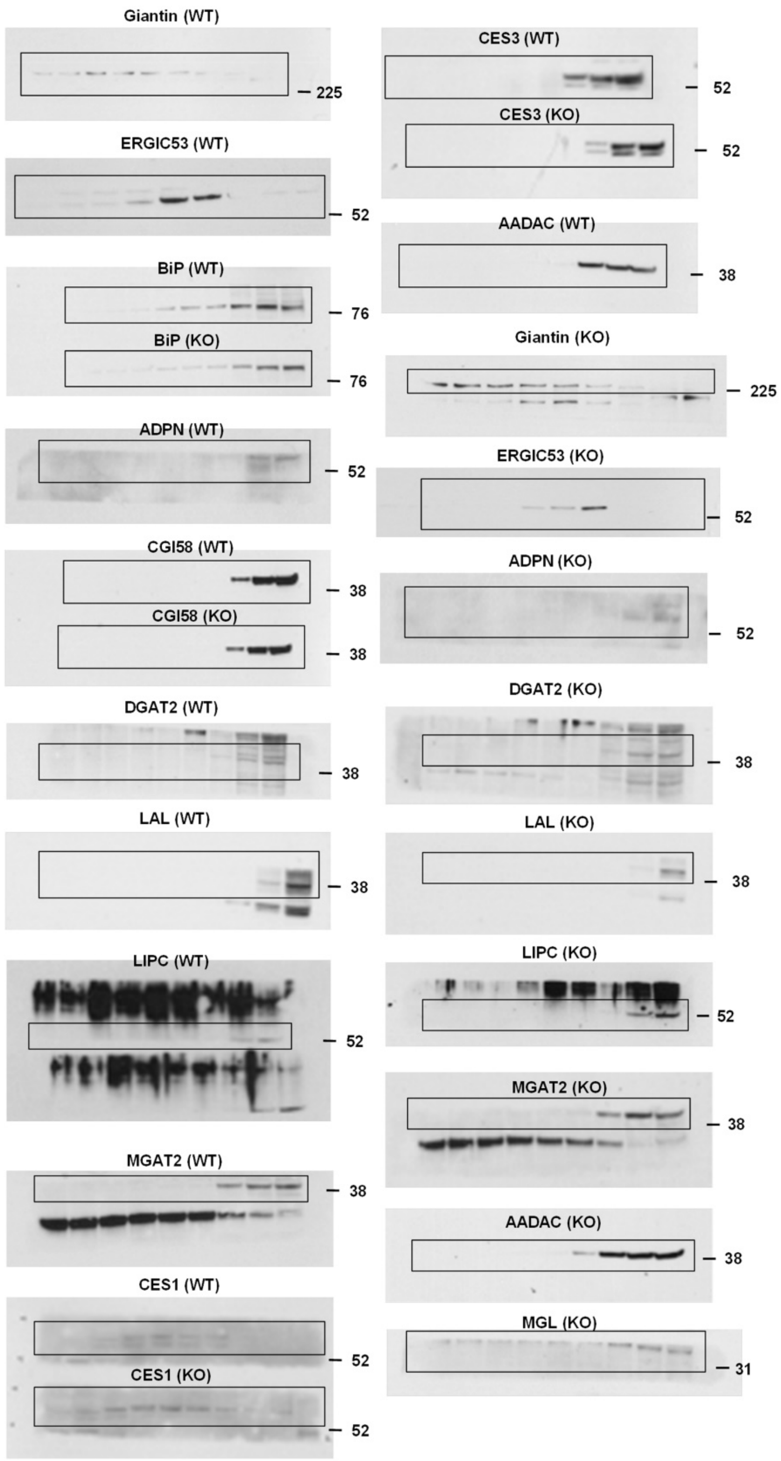

Supplementary Fig. 22 | Unprocessed images (continued)

Supp. Fig. 11e

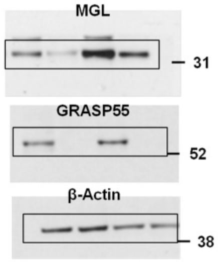

Supp. Fig. 12a

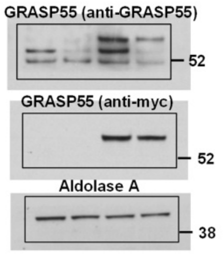

Supp. Fig. 14a

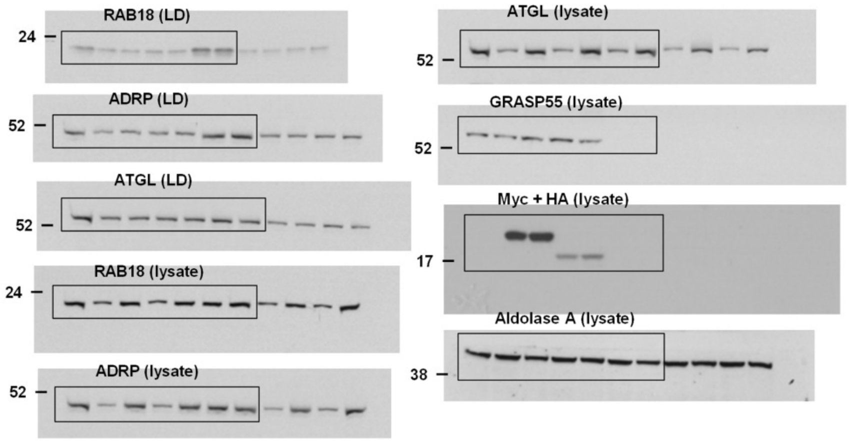

Supp. Fig. 15a

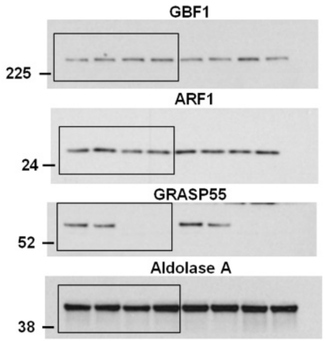

Supp. Fig. 16a

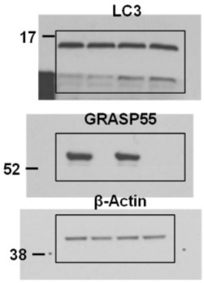

Supp. Fig. 17a

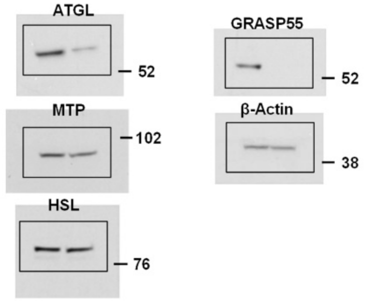

Supplementary Fig. 22 | Unprocessed images (continued)

Supplementary Table 1 | Genotype distribution of *Grasp55*$^{-/-}$ mice at birth.

Grasp55	+/+	+/-	-/-
Expected	25.0%	50.0%	25.0%
Actual (n=341)	25.2% (86)	52.2% (178)	22.6% (77)

Supplementary Table 2 | Organ weight of *Grasp55*$^{+/+}$ and *Grasp55*$^{-/-}$ mice.

	Grasp55$^{+/+}$	Grasp55$^{-/-}$	Grasp55$^{+/+}$	Grasp55$^{-/-}$
	Weight (g)	Weight (g)	Relative Weight (% per body weight)	Relative Weight (% per body weight)
White Adipose Tissue	0.072 ± 0.004	0.009 ± 0.005**	0.500 ± 0.029	0.095 ± 0.052**
Brown Adipose Tissue	0.035 ± 0.008	0.018 ± 0.003*	0.241 ± 0.056	0.184 ± 0.036
Liver	0.750 ± 0.114	0.425 ± 0.093*	5.227 ± 0.796	4.442 ± 0.968
Kidney	0.234 ± 0.026	0.140 ± 0.023**	1.631 ± 0.178	1.464 ± 0.243
Intestine	1.051 ± 0.040	0.717 ± 0.047**	7.324 ± 0.279	7.482 ± 0.493
Colon	0.251 ± 0.015	0.151 ± 0.012**	1.748 ± 0.102	1.577 ± 0.125
Lung	0.134 ± 0.004	0.075 ± 0.019**	0.936 ± 0.027	0.787 ± 0.195
Heart	0.121 ± 0.015	0.066 ± 0.009**	0.840 ± 0.102	0.694 ± 0.098
Skeletal Muscle	0.167 ± 0.021	0.113 ± 0.019*	1.164 ± 0.145	1.183 ± 0.197

Organ weight was measured at age of 4 weeks (n = 6). *$p < 0.05$, **$p < 0.01$, relative to *Grasp55*$^{+/+}$ (Student's *t* test). Source data are provided as a Source Data file.

Supplementary Table 3 | Plasma lipid and protein levels of *Grasp55*$^{+/+}$ and *Grasp55*$^{-/-}$ mice.

	Grasp55$^{+/+}$	Grasp55$^{-/-}$
Triglycerides (mg/dL)	79.00 ± 2.85	63.28 ± 3.48**
Total Cholesterol (mM)	2.63 ± 0.10	1.69 ± 0.09**
Albumin (g/dL)	2.65 ± 0.40	2.88 ± 0.28
Total Bilirubin (mg/dL)	0.65 ± 0.30	1.03 ± 0.06
Total Protein (g/dL)	5.73 ± 0.53	5.45 ± 0.07

Blood samples were taken at 12 weeks of age (n = 10). **$p < 0.01$, relative to *Grasp55*$^{+/+}$ (Student's *t* test). Source data are provided as a Source Data file.

Supplementary Table 4 | Composition of normal and high-fat diet.

Nutrient	Normal Diet<br>g% (kcal%)	High-fat Diet<br>g% (kcal%)
Protein	19.2 (20)	26.2 (20)
Carbohydrate	67.3 (70)	26.3 (20)
Fat	4.3 (10)	34.9 (60)
Kcal / g	3.85	5.24

Normal diet (Cat. No. D12450B) and high-fat diet (Cat. No. D12492) were purchased from Research Diets (New Brunswick, NJ, USA).

Supplementary Table 5 | Antibodies used in this study.

Name	Catalog number (Manufacturer)	Dilution (Use)
Primary Antibody		
anti-AADAC	MBS2526318 (MyBioSource)	1:1,000 (WB)
anti-ADPN	NBP1-00227 (Novus)	1:1,000 (WB)
anti-ADRP	LS-C3561 (LSBio)	1:100 (IHC)
anti-Albumin	4929 (Cell Signaling)	1:10,000 (WB)
anti-Aldolase A	sc-12059 (Santa Cruz)	1:1,000 (WB)
anti-APOA1	ab33470 (Abcam)	1:2,000 (WB)
anti-APOA4	ab59036 (Abcam)	1:1,000 (WB)
anti-APOB	ab31992 (Abcam)	1:500 (WB)
anti-APOB	Ab20737 (Abcam)	1:100 (IHC)
anti-ARF1	ab183576 (Abcam)	1:2,000 (WB)
anti-ARFRP1	ab108199 (Abcam)	1:2,000 (WB)
anti-ATGL	2138 (Cell Signaling)	1:1,000 (WB), 1:100 (IF)
anti-ATGL	MBS420222 (MyBioSource)	1:200 (IHC)
anti-β actin	sc-1616 (Santa Cruz)	1:1,000 (WB)
anti-BiP	3183 (Cell Signaling)	1:500 (WB)
anti-Calreticulin	ab92516 (Abcam)	1:2,000 (WB)
anti-CES1	PA5-19740 (Thermo Fisher)	1:1,000 (WB)
anti-CES3	MBS767849 (MyBioSource)	1:1,000 (WB)
anti-CGI58	ab18739 (Abcam)	1:1,000 (WB)
anti-CIDEB	ab9403 (Abcam)	1:1,000 (WB)
anti-DGAT2	NBP1-71701 (Novus)	1:1,000 (WB)

anti-ERGIC53	ab125006 (Abcam)	1:1,000 (WB)
anti-GBF1	NBP1-06526 (Novus)	1:1,000 (WB)
anti-Giantin	ab93281 (Abcam)	1:200 (WB)
anti-GM130	ab52649 (Abcam)	1:1,000(WB), 1:100 (IHC)
anti-Golgin45	NBP2-15596 (Novus)	1:500 (WB)
anti-Golgin45	PA5-41466 (Thermo Fisher)	1:100 (IHC)
anti-Golgin97	A-21270 (Thermo Fisher)	1:1,000 (WB)
anti-Golgin97	ab84340 (Abcam)	1:100 (IHC)
anti-GRASP55	ab74579 (Abcam)	1:500 (WB)
anti-GRASP55	ab204335 (Abcam)	1:50 (IF)
anti-GRASP55	sc-365602 (Santa Cruz)	1:200 (IP)
anti-GRASP65	ab30315 (Abcam)	1:1,000(WB), 1:200(IHC)
anti-HA	2367 (Cell Signaling)	1:2,000 (WB)
anti-His	sc-8036 (Santa Cruz)	1:5,000 (WB)
anti-HSL	4107 (Cell Signaling)	1:1,000 (WB)
anti-LAL	NBP1-54155 (Novus)	1:1,000 (WB)
anti-LC3	2775 (Cell Signaling)	1:1,000 (WB)
anti-LIPC	PA5-36962 (Thermo Fisher)	1:1000 (WB)
anti-MGAT2	LS-C98664 (LSBio)	1:1,000 (WB)
anti-MGL	ab24701 (Abcam)	1:2,000 (WB)
anti-MGL	ab77398 (Abcam)	1:200 (IHC)
anti-MTP	LS-C144891 (LSBio)	1:1,000 (WB)
anti-Myc	2276 (Cell Signaling)	1:2,000 (WB)
anti-Myc (Sepharose Bead Conjugate)	3400 (Cell Signaling)	1:20 (IP)

anti-Myc (Janelia Fluor® 549)	NB600-335JF549 (Novus)	1:100 (IHC)
anti-Na/K ATPase	ab7671 (Abcam)	1:2,000 (WB)
anti-normal mouse IgG	sc-2025 (Santa Cruz)	1:200 (IP)
anti-phospho-HSL	4139 (Cell Signaling)	1:1,000 (WB)
anti-RAB18	ab119900 (Abcam)	1:1000 (WB)
anti-TGN38	AHP1597 (BIO-RAD)	1:2,000 (WB)

Secondary Antibody		
anti-goat, HRP conjugated	31402 (Thermo Fisher)	1:2,000 (WB)
anti-mouse, HRP conjugated	31430 (Thermo Fisher)	1:2,000 (WB)
anti-rabbit, HRP conjugated	31460 (Thermo Fisher)	1:2,000 (WB)
anti-rabbit, HRP conjugated (Rabbit TrueBlot)	18-8816-33 (Rockland)	1:1,000 (WB for IP)
anti-mouse, HRP conjugated (Mouse TrueBlot ULTRA)	18-8817-33 (Rockland)	1:1,000 (WB for IP)
Alexa 488, anti-rabbit	A21206 (Thermo Fisher)	1:200 (IHC, IF)
Alexa 568, anti-rabbit	A10042 (Thermo Fisher)	1:200 (IHC, IF)
Alexa 594, anti-guinea	A11076 (Thermo Fisher)	1:200 (IHC)
Alexa 488, anti-goat	A11055 (Thermo Fisher)	1:200 (IHC)

WB, Western blot. IP, Immunoprecipitation. IHC, Immunohistochemistry. IF, Immunofluorescence.
